# LncRNA expression profile during autophagy and *Malat1* function in macrophages

**DOI:** 10.1371/journal.pone.0221104

**Published:** 2019-08-19

**Authors:** Zhanbing Ma, Jing Zhang, Xiangrong Xu, Yuliang Qu, Hui Dong, Jie Dang, Zhenghao Huo, Guangxian Xu

**Affiliations:** 1 Ningxia Key Laboratory of Clinical and Pathogenic Microbiology, General Hospital of Ningxia Medical University, Yinchuan, China; 2 Department of Medical Genetic and Cell Biology, College of Basic Medicine, Ningxia Medical University, Yinchuan, China; 3 Key Laboratory of Fertility Preservation and Maintenance (Ningxia Medical University), Ministry of Education, Yinchuan, China; 4 Department of Medical Laboratory, College of Clinical Medicine, Ningxia Medical University, Yinchuan, China; University of Alabama at Birmingham, UNITED STATES

## Abstract

Long noncoding RNAs (lncRNAs) are a class of functional non-coding transcripts that are longer than 200 nt and regulate gene expression via diverse mechanisms in eukaryotes. In fact, they have emerged as critical epigenetic and transcriptional regulators of autophagy in mammals in response to various stressors. Autophagy not only plays a crucial role in maintaining cellular homeostasis, but it is also essential to immunity, targets intracellular pathogens for degradation, modulates inflammation, and participates in adaptive immune responses. However, the expression profile of lncRNA and its role in regulating autophagy in macrophages have been poorly defined. Here, we used transcriptomic and bioinformatics to analysis LncRNA expression profile during autophagy and functional studies to evaluate the function of the metastasis-associated lung adenocarcinoma transcript-1 (*Malat1*) lncRNA in macrophages. A total of 1112 putative lncRNAs (240 novel lncRNAs) were identified, including 831 large intergenic, 129 intronic, and 152 anti-sense lncRNA, of which 59 differentially expressed transcripts exhibited a greater than 1.5-fold change under different conditions. The interaction of *Malat1* lncRNA with microRNA (*mir*)*-23-3p* and lysosomal-associated membrane protein 1 (*Lamp1*) was found, *Malat1* releases inhibition of *Lamp1* expression in macrophages through competitive adsorption of *mir-23-3p*. The results of this study provide a better understanding of lncRNA function in macrophages and a basis for further investigation into the roles and mechanisms of ncRNA in immunology, particularly the functions of *Malat1* and *mir-23-3p* in the pathogenesis of macrophages.

## Introduction

Protein-coding sequences only take up a very small fraction (~1.5%) of the human genome. many Non-coding sequences that are once considered as DNA junk are recently found to be essential for gene regulation by the Encyclopedia of DNA Elements project. Also, non-coding RNAs (ncRNAs) have nearly 58,648 different transcripts in humans [1]. Many ncRNAs in the genome play key roles in regulating gene expression at the transcriptional and post-transcriptional levels [2]. These ncRNAs have housekeeping, regulatory, and unknown functional roles [3]. Regulatory ncRNAs are usually classified into small ncRNAs and long ncRNAs (lncRNAs) according to their transcript lengths [4]. LncRNAs are a large class of transcribed RNA molecules of more than 200 nucleotides (nt) in length that lack of protein encoding potential and function [5]. LncRNA can be classified into different subtypes (antisense, intergenic, overlapping, intronic, bidirectional, and processed) according to the position and direction of gene transcription [6]. LncRNAs regulate biological activities through various mechanisms, including signaling [7], scaffolds [8], decoys [9]and endogenous competitive RNAs (CeRNA) [10,11]. In particular, the lncRNA-microRNA (miRNA)-mRNA CeRNA ternary network may play a key role in gene regulation [12]. Numerous studies have indicated that lncRNAs play critical roles in a wide range of biological processes, including cell differentiation and development [13,14], X-chromosome inactivation [15], the development of neurological diseases [16], cancer progression [17], and the immune response[18,19].

Autophagy is a lysosome-mediated evolutionarily conserved catabolic process in cells that serves to decompose unwanted cytoplasmic content, degenerate organelles and pathogens for high-throughput chemical recycling, and regulate cellular functions under normal and stressed conditions [20]. Similar to macrophages and dendritic cells, autophagy in antigen-presenting cells (APCs) not only acts as a phagocyte, but also plays a crucial role in initial immunity [21]. Increasing evidence on the fundamental role of autophagy in pathogenic microorganisms and cell host interactions has emerged [22]. Macrophages are part of the mononuclear phagocytic system and play crucial roles in the nonspecific immune response through autophagy. Thus, understanding how lncRNA regulates autophagy in macrophages may help to understand pathogen invasion and survival.

Accumulating data have revealed that many aspects of the autophagy process are regulated by lncRNAs [23–25]. Specifically, one of the mechanisms by which lncRNAs regulate cellular functions is by acting as CeRNA [26,27]. For example, the autophagy-promoting factor lncRNA regulates autophagy and myocardial infarction by functioning as a CeRNA sponge for *mir-188-5p* [28]. In addition, the metastasis-associated lung adenocarcinoma transcript-1 (*Malat1*) lncRNA also functions as a CeRNA to regulate autophagy. *MALAT1* is highly conserved lncRNA, which reported as a nuclear-retained in mammals, also known as *NEAT2*. It has been reported that *MALAT1* promotes gene activation or inhibition in a cell type-specific manner by promoting specific chromatin regulators, and it is likely to be used as a scaffold to recruit proteins in the vicinity of nuclear plaques and exert gene regulation. Recently, some studies also have demonstrated that *Malat1* interacts with the RNA-binding protein human antigen R, and silencing of *Malat1* greatly enhances the post-transcriptional regulation of *TIA-1*, further inhibiting autophagy [29]. The *Malat1* lncRNA is a potent autophagy inducer that protects brain microvascular endothelial cells against oxygen-glucose deprivation/re-oxygenation-induced injury by sponging *miR-26b* and upregulating the expression of *unk-51-like autophagy activating kinase 2* [30]. Moreover, *Malat1* activates autophagy and upregulates *stathmin*, *Ras-related protein Rab-5A*, and *autophagy related 4D* expression in glioma through cavernously adsorption of *miR-101*, promoting cell proliferation [31]. LncRNA *Malat1* expression is upregulated in LPS-activated macrophages, while it is downregulated in IL-4 activated macrophages [32]. In summary, *Malat1* is involved in cell autophagy, immunology and may play a sponge in the RNA triplet network consisting of lncRNA-miRNA-mRNA.

Macrophages are important APCs and the main cells of the phagocyte system. However, the expression and function of lncRNAs in macrophages during autophagy are still largely unknown. In this study, we focused on determining the expression profiles of lncRNA and mRNA in murine RAW264.7 macrophages treated under different conditions to induce or inhibit autophagy. Among the differentially expressed lncRNAs (DELs), The possible sponge mechanism of *Malat1* lncRNA in regulating autophagy in macrophages was further explored and discussed. The results of this study provide the differential expression profiles of lncRNAs during autophagy, and we confirm that *Malat1* lncRNA function as a CeRNA to regulate the expression of lysosomal-associated membrane protein 1 (*Lamp1*) by sponging *mir-23-3p* in macrophages.

## Materials and methods

### Materials

#### Cell culture

The RAW264.7 cell lines (obtained from ATCC, Beijing, China) were cultured in Dulbecco’s modified Eagle’s medium (DMEM, Thermo Fisher Scientific, USA) supplemented with 10% fetal bovine serum (FBS, Gibco) at 37°C with 5% CO_2 (_vol/vol).

#### Treatments

The autophagy inducer RAPA and 3-MA were dissolved respectively in DMSO or double-distilled water at stock concentrations of 10 mM. The cells were plated in 6-well plates at the appropriate cell density. When confluence reached about 50–70%, cells were treated with 3-methyladenine (3mM, 12h) or rapamycin (50nm, 2h) and STV in HANKs buffer for 6h before harvesting cells to acquire total RNA.

### Methods

#### High-throughput sequencing

Total RNA quality was checked with electrophoresed formaldehy de-denatured agarose gel and quantified with a spectrophotometer (NanoDrop ND-2000, USA). According to the composite sequencing quality requirements, 10 μg RNA was mixed from each condition with three repeats and then submitted to Biomarker Technologies Co. Ltd. (Beijing, China) for high-throughput transcriptome sequencing. For detecting lncRNAs, the cDNA library was constructed in a ribosome-free manner according to the instructions of the Illumine Kit. All clean reads of this work are available from the Sequence Read Archive (SRA) of the National Center for Biotechnology Information (NCBI) database (PRJNA544884).

#### Pipeline for identifying lincRNAs

A computational lncRNA dig pipeline was used to detect putative lncRNAs of each condition from RNA-seq read mapping and transcriptome assembly generated according to published methods [33]. To obtain putative novel non-coding transcripts and filter out known transcripts, the unique assembled transcriptome dataset was compared to the Ensemble Mouse genome annotation by using the Cuffcompare program from the Cufflinks package. Consequently, the assemblies that matched annotations could be identified. Unknown transcripts of more than 200 nt length and one exon were selected as candidate lncRNA transcripts. Moreover, the novel transcripts were clustered into different lncRNAs types, including lincRNA, intronic lncRNA, and anti-sense lncRNA based on their locations relative to the known genes by using Cuffcompare according to the class code. The protein-coding potential of candidate lncRNAs was analyzed by integrating the results of three widespread computational coding tools, namely, CPC, CNCI, and CPAT, to maximize the prediction of true-putative lncRNAs. Finally, putative protein-coding RNAs were filtered out by using minimum length, exon number threshold, and Pfam blast.

#### Differential expression analysis

For lncRNA sequencing, after library construction, RNA-seq (PE250) and the sequence read went through the data cleaning procedure. The ncRNAs among the different groups were compared as follows: the expression of four samples to obtain the FPKM was normalized, and the FC and FDR were calculated from the normalized expression. The logarithmetics FC formula: FC = Log_2_ (Treatment/Control).

#### LncRNA target prediction

Based on the functioning mode between the lncRNA and its target, we adopted two different prediction methods. The cis role of lncRNA is to act on neighboring target genes. We searched for coding genes within 100 kb upstream and downstream of lncRNA to predict putative cis target genes of lncRNA, followed by analysis of the functions of these coding genes to annotate lncRNA. Second, lncRNA and mRNA militate due to the base complementary, so we mainly used lncTar [34] to predict lncRNA targets by calculating the free energy and standardized free energy of mating sites, and below the threshold of the standardized free energy could be deemed as antisense targets of lncRNA. The GO functional and pathway analysis of the putative target genes were performed by using ClueGO. RNA22 and PITA database were used to explore the connection between lncRNA Malat1 and its potential sponge miRNA targets.

#### Total RNA extraction and qPCR analysis

After stimulating, cells were washed three times with PBS, and 2 ml TriZol reagent (Invitrogen) was added to 6-wells culture plate in each group. RNA extraction followed the manufacturer's instructions (Invitrogen). The RNA quality was checked by NanoDrop ND-2000 (Thermo Fisher Scientific, Waltham, MA, USA), and RNA integrity was assessed by standard electrophoreses in formaldehyde-denatured agarose gel. qPCR was used to confirm the lncRNA, miRNA, and mRNA expression levels. The RAW264.7 cells were treated with RAPA, and the expression levels of eight DELs, *Lamp1*, and *mir-23-3p* were measured by qPCR analysis. For lncRNAs, cDNA was synthesized using the Trans Script First-Strand cDNA Synthesis Super Mix Kit (Thermo Fisher Scientific) with random primers at 65°C for 30 min to degenerate the RNA secondary structure. For miRNAs, cDNA was synthesized using the PrimeScript™ RT reagent Kit (Takara Biotech) with specific primer (mir-23-3p stem loop primers). The qPCR reaction was performed using SYBR Green Gene Expression Master Mix (Applied Biosystems, USA) on the StepOne Plus Real-Time PCR system (Applied Biosystems) following the manufacturer's protocols. Small nuclear RNA (*U6*) was used for normalization. Glyceraldehyde three-phosphate dehydrogenase (*GAPDH*) and small nuclear RNA (*U6*) acted as endogenous controls to normalize the relative gene abundance. qPCR experiments and data analysis were determined using the relative quantification 2^-ΔΔCT^ method according to MIQE guidelines [35]. All of the primers for qPCR are shown in **S3 Table**.

#### Vector construction and cell transfection

For luciferase reporter assays, *mir-23-3p* target genes were predicted using in silico software (TargetScan and miRanda) and Malat1 sponge miRNA was obtained by RNA22 and StarBase. The DNA fragment containing the mir-23-3p MRE and MRE-Mut was amplified through PCR *in vitro* and the sequence was inserted downstream of the pmirGLO vector (Promega) of the renilla luciferase encoding gene between the restriction sites of *Nhe* I and *Sal* I. The cDNA generated from Raw264.7 as template to generate luciferase expression vector of pmirGLO-Lamp1-Wt/Mut or PmirGLO-Malat1-Wt/Mut recombinant plasmid. For autophagy flux monitor and *Malat1* overexpression assays, the fusion gene of partial CMV-MCS-mRFP-EGFP-LC3 with double restriction sites, *Not* I and *Sal* I was designed, optimized in MCS, codon bias and sensitivity to acid, synthesized through PAS (Zoonbio Biology, China), and then inserted into the PCDH-CMV-MCS-EF1a-CopGFP (SBI) vector between the same restriction sites to construct autophagy flux lentivirus expression plasmid, named *PCDH-Duo*. *Malat1* cDNA was amplified through PCR from PmirGLO-Malat1-Wt plasmids with *EcoR* I and *BamH* I restriction site, then sub-cloned into PCDH-Duo to generate PCDH-Duo-Malat1 lentivirus over-expression plasmid. All of the sequences, primers and PCDH-Duo plasmid map are shown in **S6 Fig**. Transfection of the Raw264.7 cells was performed according to instructions of the Lip3000 and P3000 Transfection Agent (Thermo Fisher Scientific). The mix of Malat1-siRNA and ASO was bought from Riboio (Sheng Zheng, China). *Mir-23-3p* mimics, inhibitor, and relevant NC or NC-FAM were bought from Gene Pharma (Shanghai, China). All of the vector primer and RNA oligo in this work are shown in S3 Table.

#### Lentivirus production

For *Malat1* overexpression, the 600 bp fragment containing *mir-23-3p* MRE of *Malat1* complementary DNA with *EcoR* I and *BamH* I restriction sites was sub-cloned into the PCDH-Duo lentivirus vector (Laboratory construction, information of vector was shown in **S6 Fig**) and co-transfected with plp1, plp2, and vsv.g into 293FT cells to generate PCDH-Duo-Malat1 lentivirus. PCDH-Duo empty lentivirus construct (Laboratory construction) was used as a negative control for *Malat1* overexpression. Lentivirus production was conducted according to the manufacturer’s instructions of lip3000.

#### Dual luciferase report assays

The Raw264.7 cells were cultured in 96-well plates and co-transfected with 50 nM mimics, 100 nM inhibitor or relevant control *mir-23-3p* (Gene Pharma), and luciferase expression vector carrying wild-type or mutant 3’-UTR of *Lamp1*, wild-type or mutant Malat1 fragment containing MRE using Lipofectamine 3000 (Invitrogen). At 48 h after transfection, relative renilla luciferase activity was detected using the dual luciferase assay kit (E2910, Promega, Madison, WI) according to the manufacturer’s instructions, and the activity of firefly luciferase was the internal reference. The transfection was repeated in three independent experiments.

#### Immunofluorescence

After cell transfection for 6 h, the slides of Raw264.7 cells were washed with pre-warmed (37°C) 1× PBS three times, 5 min each. Then, the specimens were fixed in 4% paraformaldehyde (pH 7.5) at room temperature for 30 min. The cells were rinsed three times with 1× PBS for 10 min each. To each group of specimens, 0.2% Triton X-100 was added, followed by incubation at room temperature for 5 min to fully permeate the cell membrane. Then, the cells were rinsed again and blocked at room temperature for 30 min with 5% BSA. After overnight incubation at 4°C with primary LC3 antibody at a dilution of 1:100 (Proteintech), the cells were incubated with FITC-labeled goat anti-mouse secondary antibodies (Beyotime) at 37°C for 80 min after washing with PBS containing 0.05% Tween 20 (PBS-T). The cells were then washed again 5 times for 10 min with PBST, and the nuclei were counterstained with DEPI (Beyotime) for 5 min. Then, cells were visualized under a LSCM (Olympus FV-1000, Tokyo, Japan) using FV10-ASW software. At least triple independent experimental group were performed.

#### RNA-FISH

The Raw264.7 cells were cultured on slides placing in 24-well plates at appropriate density (~6 × 10^4^/well) until 50% convergence reached. The cells were washed with pre-warmed (37°C) 1× PBS three times for 5 min each, fixed in 4% paraformaldehyde at room temperature for 10 min, rinsed again, and incubated with pre-chilled penetrant at 4°C for 5 min. The permeabilization solution was discarded and the cells were rinsed with PBS three times for 5 min each. Then, 200 μL prehybridization solution was added to each well and incubated at 37°C for 30 min, after which 2.5 μL of 20 μM Malat1 RNA-FISH probe mix (Riboio) or reference FISH probe Mix (18S/U6, Riboio) was added to 100 μL hybridization solution in each well and incubated overnight at 37°C. The cells were washed with Hybrid fluid I/II/III successively three times for 5 min each. The nuclei were counterstained with DAPI (Beyotime) for 5 min. Then cells were visualized under an LSCM (FV-1000; Olympus, Tokyo, Japan) using FV10-ASW software. At least triple independent experimental group were performed.

#### Western blot analysis and WES

Cells were harvested and lysed in RIPA buffer containing protease inhibitors, and the total cell collected. Proteins were separated by 7.5%, 10%, or 15% SDS-polyacrylamide gel electrophoresis and electro transferred to Hybond membranes (Amersham, Munich, Germany). BSA (5%) was used to block membranes for 2 h at room temperature. After blocking, primary antibodies targeting ATG5, P62 (SQSTM1), LAMP1 (1: 1000, Proteintech Group, USA), LC3 (1: 2000, CST, MA, USA), and β-actin (1:5000; Proteintech Group, Chicago, IL, USA) were incubated with the blot overnight at 4°C. The following day, secondary antibodies were added for 2 h at room temperature after the membrane was washed three times with TBST. Protein was visualized using an enhanced chemiluminescence system according to the manufacturer’s protocol (Santa Cruz Biotechnology, California, CA, USA). Furthermore, WES was used to detect LAMP1 expression in the rescue experiment according to the manufacturer protocols (SM-W004—Protein Simple, San Jose, CA) using a 12–230 kDa WES separation module coupled to a 25-capillary cartridge. The WES system is not a typical immunoblot method where qualitative images are quantified to generate data. Rather, chemiluminescence is used to detect and directly quantify the data.

#### Statistical analysis

Statistical analyses were performed using SPSS 19.0 (SPSS, Chicago, IL, USA) and the R platform. All of the experiments were repeated at least three times. Data are expressed as the mean ± standard deviation (SD). Two-tailed student’s *t* test or one-way analysis of variance was used to analyze the statistical difference between different treatments. *P* < 0.05 was considered statistically significant.

## Results

### Systematic identification of lncRNA in Raw264.7 cells

**Fig 1:**
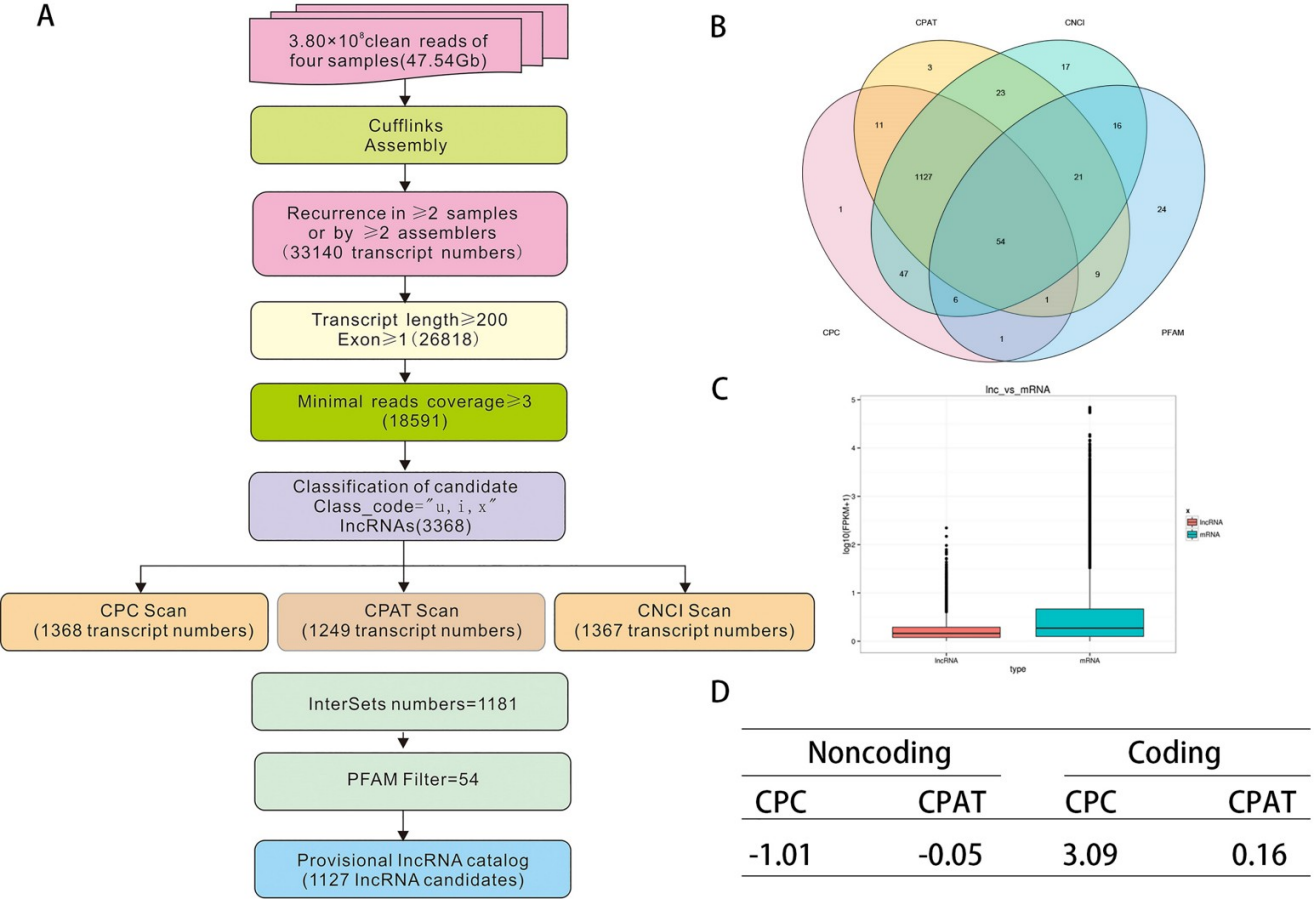
An integrative techflow and computational pipeline for the systematic identification of LncRNAs in Raw264.7. (A) Informatics pipeline for the identification of LncRNAs in Raw264.7. (B) Venn chart showing the numbers of candidate LncRNAs filtered by the CNCI, CPC (Coding Potential Calculator), CPAT, and PFAM with the default parameters. (C) Box plots of log10 maximum expression values (FPKM+1) for protein-coding (Blue) and lncRNA (red) genes. Boxes represent first and third quartiles. Whiskers are 1.5-times the interquartile range. (D) Average score of Noncoding and Coding sequences calculated with CPC and CPAT.

We performed RNA-seq to identify lncRNAs in Raw264.7 cells under different stressors including negative control (NC, dimethyl sulfoxide [DMSO]), 50 nM RAPA 12h, 3 mM 3-MA 6h and amino acid-deprived (starvation [36]) conditions 6h. After quality control (**S1 Table**), the reliable sequencing data as input for downstream identification. To effectively distinguish between protein-coding and non-coding sequences, coding potential filtering was performed according to an integrative techflow and computational pipeline (**Fig 1A**). In this way, 1112 lncRNAs were ultimately obtained (**Fig 1B**). The fragments per kilobase of transcript per million (FPKM) was used to represent the abundance of lncRNAs. In accordance with a previous report [37], we observed that the average lncRNA expression level under different conditions was lower than protein-coding gene expression (**Fig 1C**). Analysis using computational coding tools showed that noncoding sequences from the unknown transcript had an average CPC score of -1.01 and CPAT score of -0.05 compared with coding sequences, which had scores of 3.09 and 0.06, respectively (**Fig 1D**). All of the information on the lncRNAs identified by our pipeline is listed in **S2 Table**.

### Characterization of lncRNAs in Raw264.7 cells

**Fig 2:**
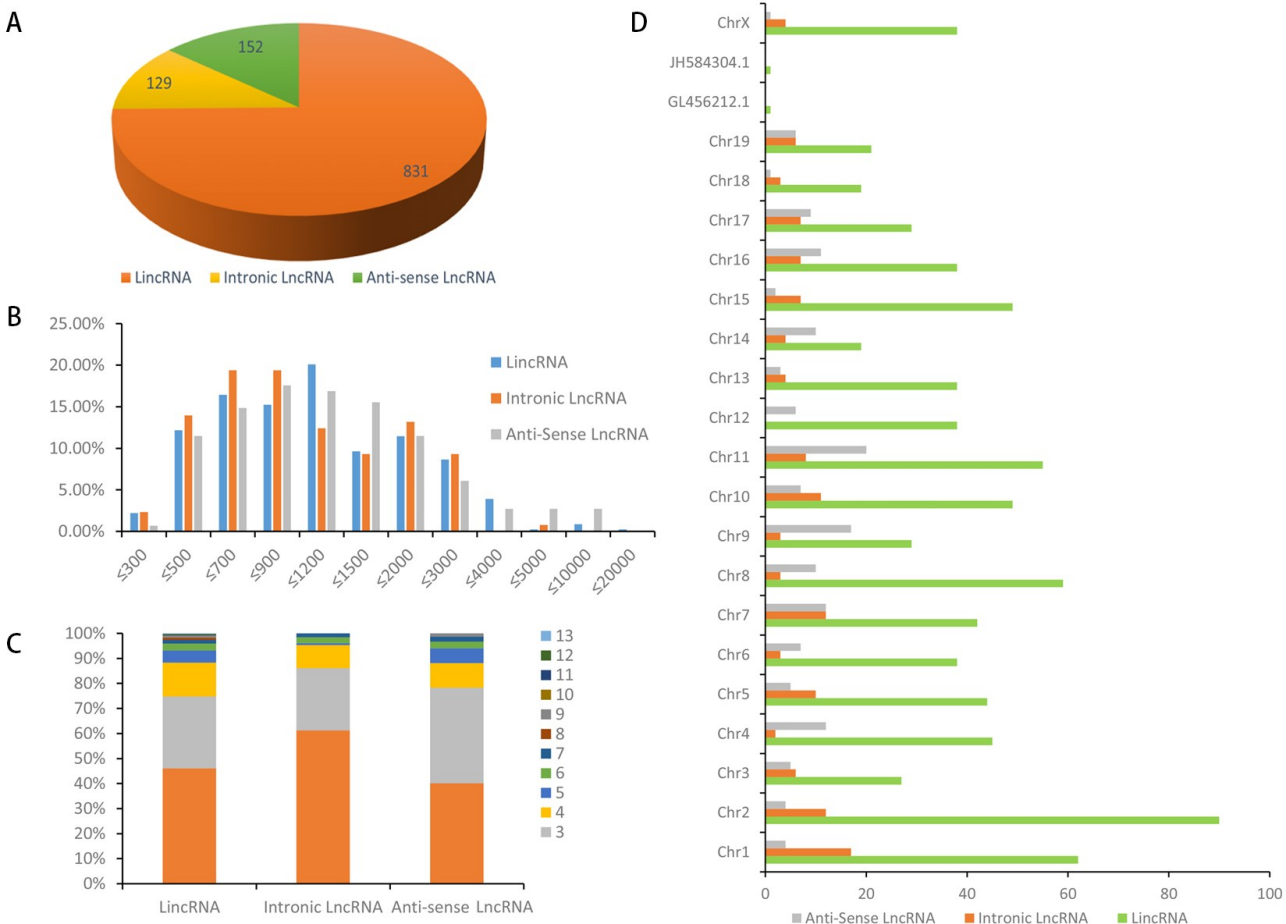
Characteristics of Raw264.7 lncRNAs. (A) Numbers of lincRNAs, intronic lncRNAs and antisense; ncRNAs in Raw264.7. (B) Transcript length distribution of lincRNAs, intronic lncRNAs and antisense lncRNAs. (C) The number of exons per transcript for LncRNAs. (D) Distribution of lincRNAs, intronic lncRNAs and anti-sense along each chromosome.

For the first time, the characteristics and transcription patterns of Raw264.7 lncRNAs were investigated in our study. A total of 1112 lncRNAs in Raw264.7 cells were identified, including 831 lincRNAs, 129 intronic lncRNAs, and 152 anti-sense lncRNAs (**Fig 2A**). Of these lncRNA, 240 were novel, including 158 lincRNAs, 60 intronic lncRNAs, and 22 anti-sense lncRNAs. LincRNAs comprised the major part of total Raw264.7 lncRNAs (75%). Full-length Raw264.7 lncRNA transcripts (median length: 953 nt) were longer than human lncRNA transcripts (median length: 592 nt). Interestingly, almost all of the lincRNA lengths were shorter than 4000 nt, and 90% of intronic RNAs were shorter than 3000 nt in Raw264.7 cells. However, less than 20% of Raw264.7 lincRNAs and anti-sense lncRNAs were shorter than 500 nt. Approximately 75% of the intronic lncRNAs ranged from 500 to 3000 nt, whereas only about 5% of lncRNAs were longer than 3000 nt (**Fig 2B**). The exon numbers in different types of lncRNAs of Raw264.7 were compared. LincRNA had 13 exons, intronic lncRNA had 7, and anti-sense lncRNA had 9. Among all of the lncRNAs, 45.97% of lincRNAs, 61.24% of intronic lncRNAs, and 40.13% of anti-sense lncRNAs had two exons. Exon numbers of 2–4 accounted for 90% of all of the exon numbers (average: 90.57%; **Fig 2C**). LncRNAs were evenly distributed in each chromosome, and lincRNAs did not show a significant chromosome location preference (**Fig 2D**). Properties, such as transcript abundance, lengths, exon number, and open reading frame (ORFs) of lncRNAs and mRNAs in Raw264.7, were also compared under the same conditions (**S1 Fig**).

### DEG profiling of lncRNAs in macrophages

**Fig 3:**
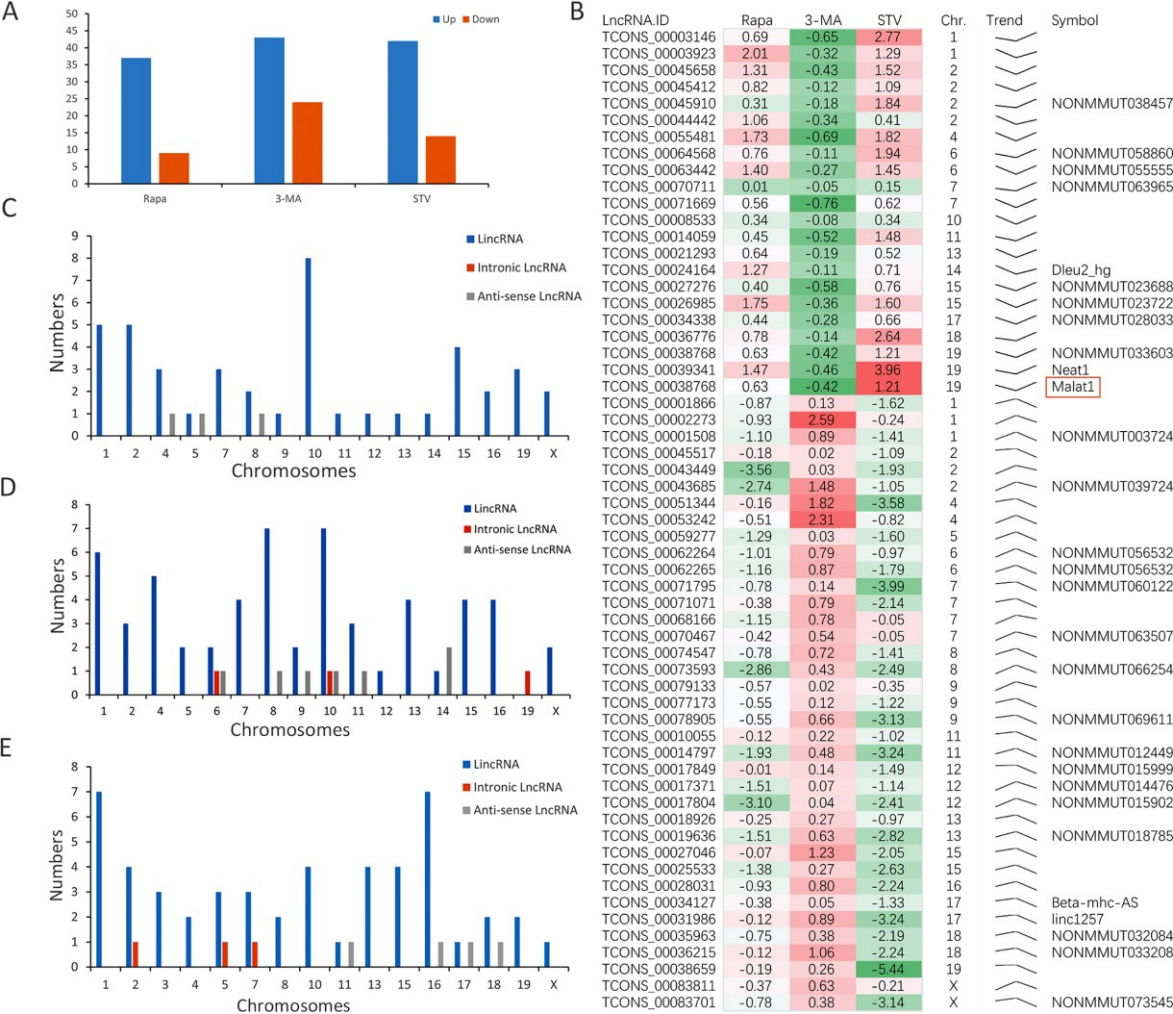
LncRNA expression file. (A) The numbers of DEL under each condition. (B) Two expression clusters containing 59 DELs. (C-E) Different type and chromosome distribution of DEL in each group (C: 50 nM Rapa, D: 3 mM 3-MA, E: STV).

The lncRNA expression profiles under conditions of RAPA, 3-MA, and STV were compared. In order to improve the accuracy of the screening, based on an FPKM ≥ 0.5, DELs were identified by P ≤ 0.05 using Fisher’s exact test, FDR ≤ 0.05, and |log_2_FC| ≥ 1.5 in either of the two groups. Furthermore, compared with the NC group, 62 lncRNAs were differentially expressed in the RAPA group (32 upregulated and 30 downregulated), 65 lncRNAs were differentially expressed in the 3-MA group (43 upregulated and 24 downregulated), and 55 lncRNAs were differentially expressed in the STV group (43 upregulated and 14 downregulated). The number of lncRNAs that were only expressed in one group was 23 in the RAPA group, 22 in the 3-MA group, and 40 in the STV group. The number of lncRNAs expressed in each groups are shown in **Fig 3A**. In all 1112 lncRNAs, 240 were novel lncRNAs that were responsive to different conditions **(S2 Table**), including 158 lncRNAs (65.6%), 60 intronic lncRNAs (24.9%), and 22 anti-sense lncRNAs (9.5%); 12 were consecutively expressed in all three conditions (RAPA, 3-MA, STV). After strict expression filtering, 59 DELs were screened out (**Fig 3B**), including 11 novel and 48 known lncRNAs. Of the 59 DELs, we identified five known lncRNAs by a BLAST search of the lncRNA database and NONCODE database. In particular *Malat1* (*Neat2*) had a huge FPKM (≥50) and at least a two FC in the RAPA and STV groups (**Table 1**), which most likely promotes autophagy. The clusters, chromosome distribution, and proportion of lncRNAs under different conditions were analyzed and the results shown there was no chromosome preference of lncRNAs under the different conditions (**Fig 3C, Rapa; Fig 3D, 3-MA and Fig 3E, STV**). More than 90% DELs in Raw264.7 cells were lincRNAs, whereas intronic and anti-sense lncRNA only accounted for a minor percentage (<10%). Hence, lincRNAs were more responsive during autophagy in our research model, while intronic and anti-sense lncRNAs were less affected.

**Table 1:** Known lncRNAs from the lncRNA and NONCODE databases.

LncRNA.Transcript.ID	FPKM					Log_2_FC			LncRNADB
	NC	Rapa	3-MA	STV	Chr.	Rapa	3-MA	STV	
TCONS_00039341	1.1168	3.0858	0.8130	17.3318	19	1.47	-0.46	3.96	Neat1
TCONS_00024164	0.7419	1.7895	0.6883	1.2134	14	1.27	-0.11	0.71	Dleu2_hg
**TCONS_00038768**	95.3854	147.3102	71.0835	220.7682	19	0.63	-0.42	1.21	Malat1(Neat2)
TCONS_00031986	0.6253	0.5744	1.1604	0.0663	17	-0.12	0.89	-3.24	Linc1257
TCONS_00034127	2.9721	2.2811	3.0819	1.1861	17	-0.38	0.05	-1.33	Beta-MHC-AS
TCONS_00003923	6.5679	26.4309	5.2733	16.0668	1	2.01	-0.32	1.29	Gas5

Bold: FPMK ≥ 5 in four groups

### LncRNA targets for pathway analysis

Cis and trans targets were used as input for pathway analysis. DAVID online software was used to analyze the statistical enrichment of DEL target genes in Kyoto Encyclopedia of Genes and Genomes (KEGG) pathways. Based on the results of significantly DEL analysis, different autophagy-responsive pathways were found under different conditions (**S2 Table**). In the RAPA group, there were four lncRNA targets involved in the phosphoinositide 3-kinase (PI3K)-Akt pathway (ENSMUSG00000004056, ENSMUSG00000021457, ENSMUSG00000023067, and ENSMUSG00000025856), two involved in the mammalian target of rapamycin (mTOR) pathway (ENSMUSG00000004056 and ENSMUSG00000028278), and five involved in the tuberculosis pathway (ENSMUSG00000004056, ENSMUSG00000007891, ENSMUSG00000013160, ENSMUSG00000018819, and ENSMUSG00000021457). In the 3-MA group, ENSMUSG00000033467 was involved in the Janus kinase-signal transducers and activators of transcription signaling pathway, and in the STV group, there were four targets (ENSMUSG00000020248, ENSMUSG00000021457, ENSMUSG00000039217, and ENSMUSG00000051439) involved in the tuberculosis pathway. Autophagy relative lncRNA-targets pair’s datasets shown in Table 2.

**Table 2:** Autophagy-related lncRNAs.

Treat	Transcript.id	LncRNA. Symbol	NC. FPKM	Treat.FPKM	FDR	log_2_FC	Targets Gene.ID	Symbol
Rapa	TCONS_00023913	-	0.0000	1.1339	1.84297E-14	6.39	ENSMUSG00000098557	Kctd12
	TCONS_00082080	NONMMUT073660	0.0000	1.9297	1.60726E-06	4.82	ENSMUSG00000063663	Brwd3
	TCONS_00055298	NONMMUT046615	0.5167	2.0777	0.007054927	1.96	ENSMUSG00000028278	Rragd
	TCONS_00053382	NONMMUT048051	0.0000	1.5544	1.03152E-05	4.62	ENSMUSG00000044303	Cdkn2a
	TCONS_00004955	NONMMUT000128	0.8223	6.9005	1.10592E-06	2.99	ENSMUSG00000025917	Cops5
	TCONS_00060740	NONMMUT054895	4.6242	0.0000	0	-7.74	ENSMUSG00000019054	Fis1
	TCONS_00038768	NONMMUT033610	95.3854	147.3102	1.00592E-06	0.63	ENSMUSG00000031447	Lamp1
3-MA	TCONS_00014193	NONMMUT011771	0.7140	0.0000	0	-7.05	ENSMUSG00000018659	Pnpo
	TCONS_00008237	NONMMUT008160	1.1633	0.0000	1.16417E-08	-5.30	ENSMUSG00000025364	Pa2g4
	TCONS_00008237	NONMMUT008160	1.1633	0.0000	1.16417E-08	-5.30	ENSMUSG00000025373	Rnf41
	TCONS_00060740	NONMMUT054895	4.6248	0.0000	0	-8.26	ENSMUSG00000019054	Fis1
	TCONS_00023913	-	0.0000	1.4805	0	7.27	ENSMUSG00000098557	Kctd12
	TCONS_00060740	NONMMUT054895	4.6248	0.0000	0	-8.26	ENSMUSG00000004846	Plod3
	TCONS_00064926	NONMMUT056532	0.7829	0.1901	0.006511504	-1.93	ENSMUSG00000018659	Pnpo
	TCONS_00023915	-	0.7297	2.5738	2.9502E-07	1.83	ENSMUSG00000098557	Kctd12
	TCONS_00014137	NONMMUT010720	0.3265	1.1308	7.68694E-05	1.78	ENSMUSG00000000753	Serpinf1
	TCONS_00004516	LincRNA-COX2	1.4150	0.3644	0.002440544	-1.87	ENSMUSG00000032487	Ptgs2
	TCONS_00008236	NONMMUT008160	0.4915	1.9999	0.000182137	1.98	ENSMUSG00000025373	Rnf41
	TCONS_00008236	NONMMUT008160	0.4915	1.9999	0.000182137	1.98	ENSMUSG00000025364	Pa2g4
	TCONS_00004955	NONMMUT000128	0.8224	3.0498	0.003987109	1.84	ENSMUSG00000025917	Cops5
	TCONS_00053382	NONMMUT048051	0.0000	3.1468	9.10383E-14	6.10	ENSMUSG00000044303	Cdkn2a
	TCONS_00038768	NONMMUT033610	95.3854	71.0835	0.000455226	-0.52	ENSMUSG00000031447	Lamp1
STV	TCONS_00008830	-	0.0000	1.1312	4.02227E-05	4.46	ENSMUSG00000075000	Nrbf2
	TCONS_00015161	NONMMUT008283	0.0000	0.7484	2.74567E-05	4.52	ENSMUSG00000020448	Rnf185
	TCONS_00061211	-	0.0000	1.4425	5.94488E-05	4.40	ENSMUSG00000029223	Uchl1
	TCONS_00061397	NONMMUT054532	0.0000	1.6107	5.94488E-05	4.40	ENSMUSG00000008348	Ubc
	TCONS_00010935	NONMMUT011771	0.9580	0.0000	0.000655226	-4.56	ENSMUSG00000018659	Pnpo
	TCONS_00027620	NONMMUT023686	0.5352	3.0793	0.003522683	2.36	ENSMUSG00000022346	Myc
	TCONS_00060740	NONMMUT054895	4.6195	0.9621	0.009364357	-2.32	ENSMUSG00000004846	Plod3
	TCONS_00060740	NONMMUT054895	4.6195	0.9621	0.009364357	-2.32	ENSMUSG00000019054	Fis1
	TCONS_00071969	-	9.5469	1.5670	0.004403232	-2.60	ENSMUSG00000066232	Ipo7
	TCONS_00004955	NONMMUT000128	0.8214	10.9721	2.65116E-06	3.49	ENSMUSG00000025917	Cops5
	TCONS_00030139	NONMMUT029200	4.2132	32.8201	3.72584E-05	2.86	ENSMUSG00000004069	Dnaja3
	TCONS_00038768	NONMMUT033610		220.7682	1.13152E-05	1.21	ENSMUSG00000031447	Lamp1

-: indicates novel lncRNAs found in this research

### Validation of the transcription levels of eight lncRNAs

**Fig 4:**
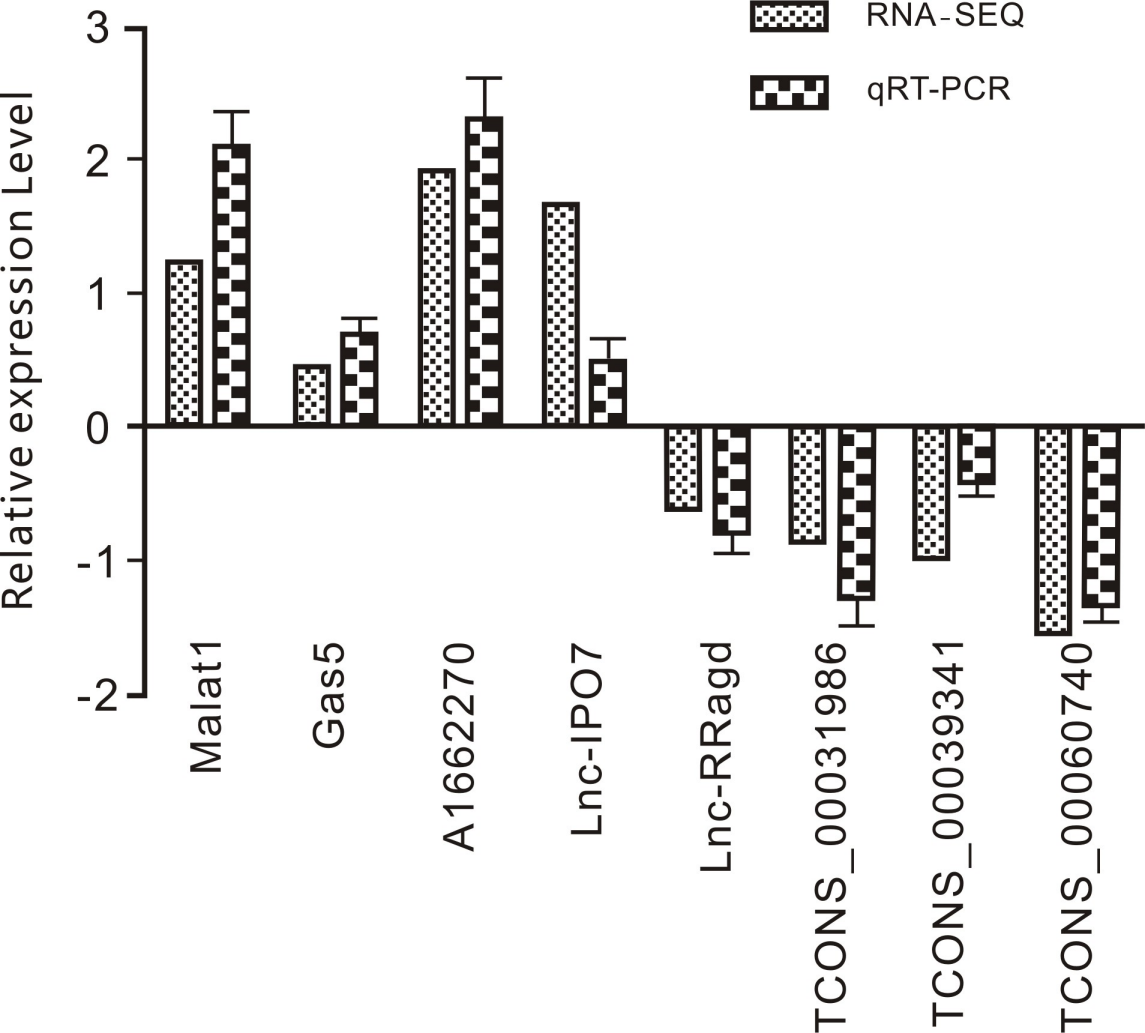
RT-qPCR validation of eight LncRNA relative expression level upon 50 nM Rapa stimulation 2 hours in Raw264.7 cell lines (Mean±SD, n = 3).

To confirm the differential expression of eight lncRNAs under Rapa condition that potentially function in autophagy, qPCR was performed (**Fig 4**). The lncRNAs were found to involved in autophagy pathways and were known lncRNAs from this work that included *Malat1*, *Gas5*, *AI662270*, *Lnc-IPO7*, *Lnc-Rragd*, *TCONS-00031986*, *TCONS-00039341*, and *TCONS-00060740*. Our qPCR results showed that the relative expression levels of the eight lncRNAs were consistent with those of RNA-seq.

### Effects of Malat1 on autophagy in macrophages

**Fig 5:**
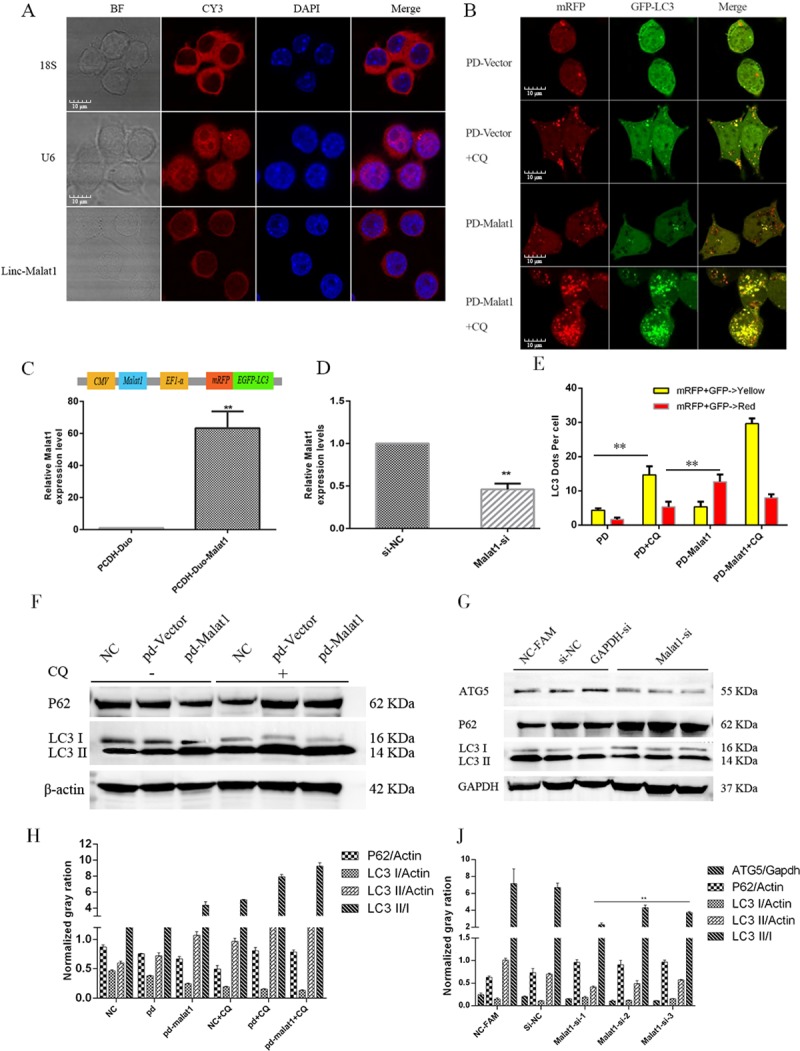
Effects of Malat1 on autophagy in macrophages. (A) RNA-FISH was performed to confirm sub cellular distribution of Malat1 in Raw264.7 cells (Scale bar = 10 μm, n = 3). (B) Confocal microscope was used to check the autophagy flux in different groups (Scale bar = 10 μm, n = 3). (C) The expression of Malat1 in Raw264.7 cells infected with PCDH-Duo-Malat1 (PD-Malat1) or vector (PCDH-Duo) Lentivirus was assessed by RT-qPCR (Mean±SD; n = 3). GAPDH was used as the endogenous control. (D) The expression of Malat1 was assessed by RT-qPCR in Raw264.7 cells transfected with Malat1-SiRNA and ASO mix (Mean±SD; n = 3). (E) Fluorescent LC3 dots per cell was quantified by Image J software, the data represent the Mean±SD of three independent experiments. (F and H) Autophagy marker P62 and LC3 were assessed by Western blot in PD-Vector or PD-Malat1 group. The expression of autophagy marker protein includes P62, LC3 was normalized against β-actin. (G and J) Autophagy marker P62, ATG5 and LC3 were assessed by Western blot in the Malat1-siRNA or Si-NC groups. The expression of autophagy marker protein includes ATG5, P62 and LC3 was normalized against GAPDH. *P<0.05 or ** P<0.01 in experiments vs. the corresponding control group.

Among the lncRNAs selected above, *Malat1* has been the most extensively studied in many cancers and may have autophagy-promoting effects. Fluorescent in situ hybridization targeting ribonucleic acid molecules (RNA-FISH) showed that *Malat1* was mainly distributed in the cytoplasm (**Fig 5A**). Autophagy flux check revealed that overexpression of *Malat1* could promote autophagy (**Fig 5B and 5E**). Lentivirus-mediated *Malat1* overexpression (**Fig 5C**) and small interfering RNA (siRNA) and antisense oligo (ASO)-mediated *Malat1* knockdown (**Fig 5D**) were performed in Raw264.7 cells. Furthermore, western blot analysis confirmed that the overexpression of malat1 (pd-Malat1) could promote microtubule-associated protein light chain 3 (LC3) conversion and an increase in LC3 II expression. After the addition of choroquine diphosphate (CQ), the accumulation of LC3 in each group increased, but accumulation of the PD-malat1 group was the most obvious (**Fig 5F and 5H**). While knockdown of *malat1* expression (Malat1-si), increased the expression of *p62*, the ratio of LC3 I/II decreased **(Fig 5G and 5J)**. *Malat1* functions as a CeRNA to regulate autophagy [38–40]. Furthermore, bioinformatics analysis of chromatin immunoprecipitation (Chip)-seq data suggested that human *Malat1* could sponge many autophagy-related miRNAs, such as hsa-mir-23-3p, which had the same miRNA response elements (MREs) in mice, as determined by a BLAST search of the miRBase database of mature miRNA (**S2 Fig**).

### Lamp1 is the direct target of *mir-23-3p* in macrophages

**Fig 6:**
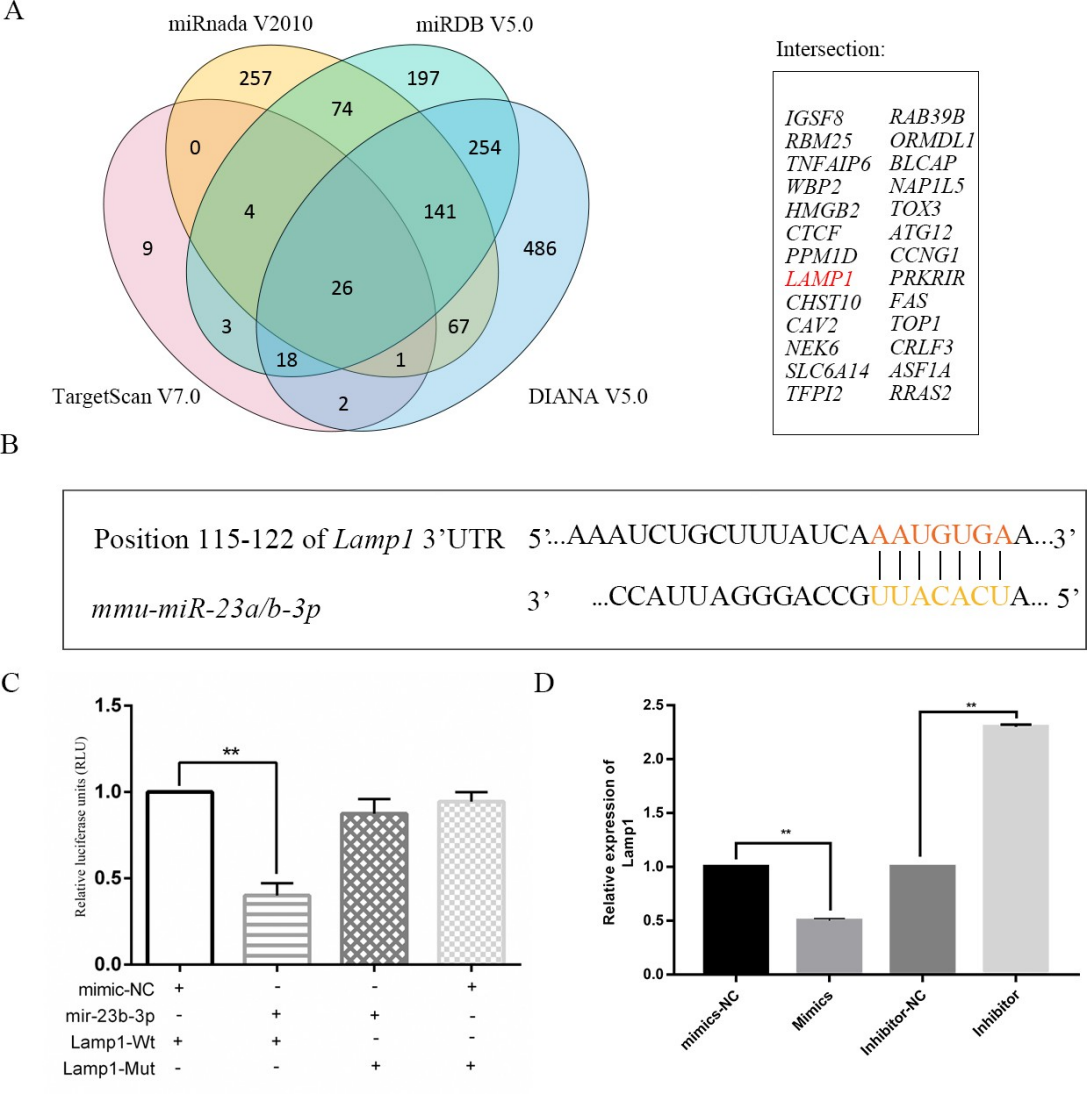
Lamp1 is a target of Mir-23b-3p in macrophage. (A) Four different databases were used to cross-predict mir-23b-3p targets and 26 potential targets presented in Venn. (B) The bioinformatics prediction of mir-23a/b-3p MRE in Lamp1. (C) Luciferase reporter assay was performed in 293T cells co-transfected with pmirGLO-Lamp1-Wt or Mut and mimics or mimics-NC in Raw264.7 cells. (D) RT-qPCR was performed to examine the expression of Lamp1 in Raw264.7 cells transfected with mimics-NC, mimics, inhibitor-NC, or inhibitor.

**Fig 7:**
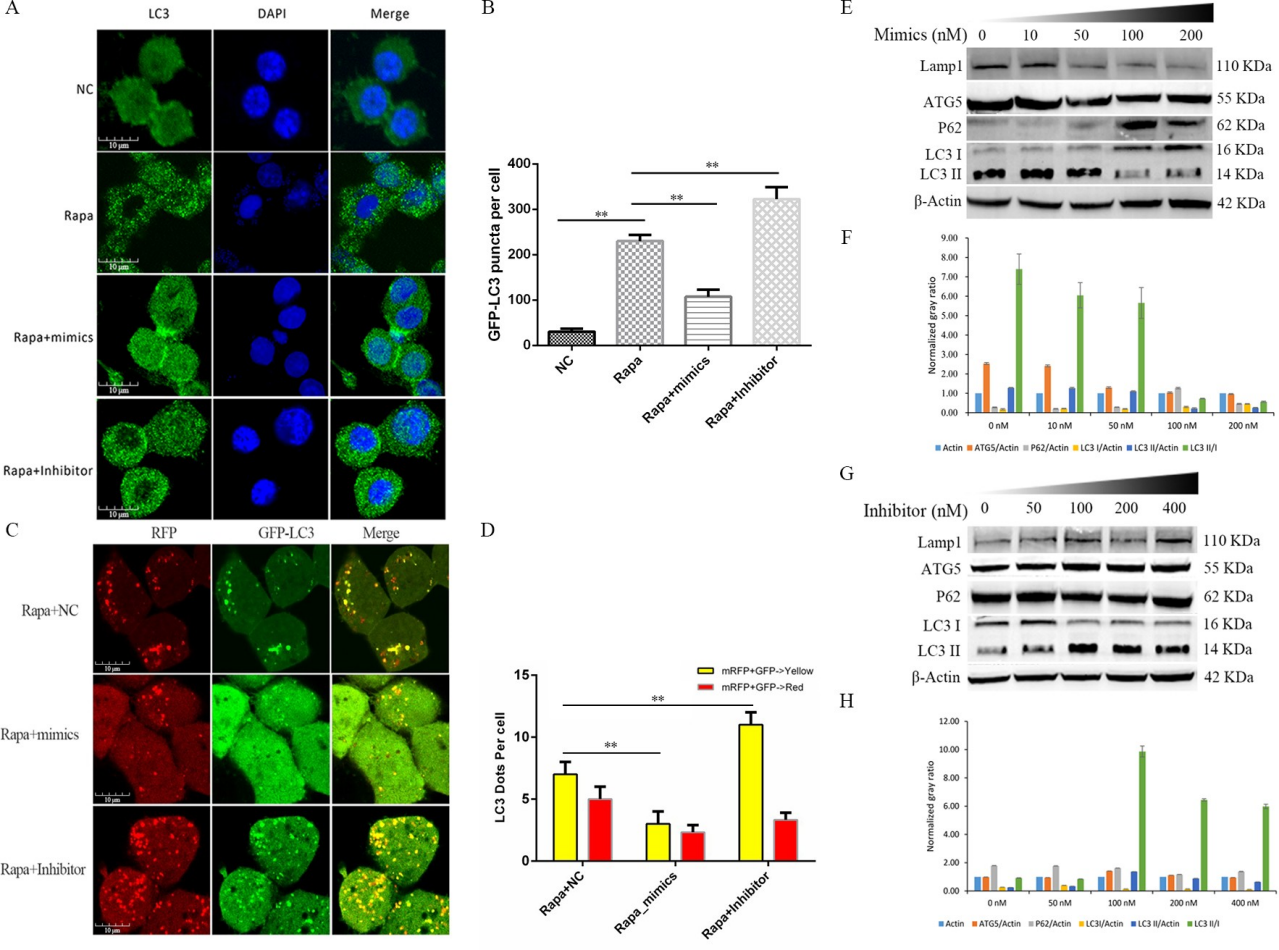
mir-23b-3p inhibits autophagy in macrophage. (A) Immunofluorescence of endogenous LC3 was checked in Raw264.7 cells under different treatment conditions (Scale bar = 10 μm). (B) GFP-LC3 puncta per cell was quantified by Image J software, the data represent the Mean±SD of three independent experiments. (C) Autophagy flux analysis was conducted in Raw264.7 cells in Rapa+NC, Rapa+mimics, or Rapa + Inhibitor group to confirm mir-23-3p function (Scale bar = 10 μm). (D) Fluorescent LC3 dots per cell was quantified by Image J software, the data represent the Mean±SD of three independent experiments. (E-H) Western blot was used to detect the protein levels of Lamp1, ATG5, P62, LC3 I/II in Raw264.7 cells treated with concentration gradients of (E) mimics (0–200 nM) or (G) inhibitors (0–400 nM). Normalized gray ratio was also compared, the expression of protein was normalized against β-actin (Mean±SD; n = 3).

*mir-23-3p* was selected from the human *Malat1* CeRNA network because it was confirmed to affect autophagy in previous reports. Four different miRNA TargetScan software programs with strict parameters were employed to determine the potential targets of *mir-23-3p* with high confidence, namely TargetScan V7.0 (Only top 100 conserved with a cumulative weighted context ^++^ score ≤ -0.4), Miranda v2010 (mirsvr_score ≤ -1.0), miRDB V5.0 (score ≥ 80), and DIANA V5.0 (miTG score ≥ 0.80). Consequently, we identified 26 mRNAs as potential targets of *mir-23-3p* (**Fig 6A**). The target of each miRNA was mapped to the protein-protein interaction (PPI) network. The results showed that *mir-23-3p* might be involved in the PI3K-Akt pathway, and co-expression of *Lamp1 and Akt1* was found (**S3 Fig**). *Lamp1* was chosen from all of the potential targets of *mir-23-3p*, depending on insilco analysis of candidate target genes and existing reports, as well as several lines of evidence supporting the fact that LAMP1 protein plays a key role in the fusion of autophagosome and lysosomes [41–43]. The *mir-23-3p* MRE was localized in 115–122 nt (**Fig 6B**) of *Lamp1* 3‘-UTR. Our dual luciferase reporter assay results suggested that *mir-23-3p* could directly target *Lamp1* 3‘-UTR (**Fig 6C and S4 Fig**). The mRNA expression of *Lamp1* was detected by qPCR, and the results indicated that overexpression of *mir-23-3p* could significantly reduce the expression of *Lamp1* in Raw264.7 cells. However, these effects were reversed in the inhibitor group (**Fig 6D**). The protein expression of LAMP1 in the cells treated with different concentrations of *mir-23-3p* mimics was detected by western blotting. The results showed that *Lamp1* was significantly downregulated in a concentration-dependent manner (**Fig 7C**), and opposite results were obtained with *mir-23-3p* inhibitors (**Fig 7E**).

### *Mir-23-3p* inhibits autophagy in macrophages

The regulatory function of *mir-23-3p* in autophagy has been reported, but whether it takes part in the autophagy of macrophage cells is still unknown. We first analyzed the expression of the endogenous autophagy marker LC3B in macrophages by immunofluorescence. when *mir-23-3p* was over-expressed (mimics) in Rapa group in macrophages could down-regulation expression of LC3B and reduced LC3B puncta. Furthermore, LC3B puncta accumulates in the RAPA group treated with mir-23b-23 inhibitor compared to the RAPA group (**Fig 7A**), which was verified by autophagy flux analysis. Moreover, autophagy flux results showed that *mir-23-3p* might regulate late autophagy due to enhanced co-localization of membrane bound red fluorescence protein (mRFP) and enhanced green fluorescent protein (EGFP) signals (**Fig 7B**). western blot analysis of macrophages treated with different concentrations of *mir-23-3p* mimics (**Fig 7C and 7D**) and inhibitors (**Fig 7E and 7F**) also showed that the endogenous autophagy markers, including autophagy related 5 (ATG5), p62, and the ratio of LC3 II/I, had a corresponding trend. These results demonstrate that *mir-23-3p* suppresses autophagy in macrophages.

### Malat1 functions as a decoy of *mir-23-3p* to regulate *Lamp1* expression in macrophages

**Fig 8:**
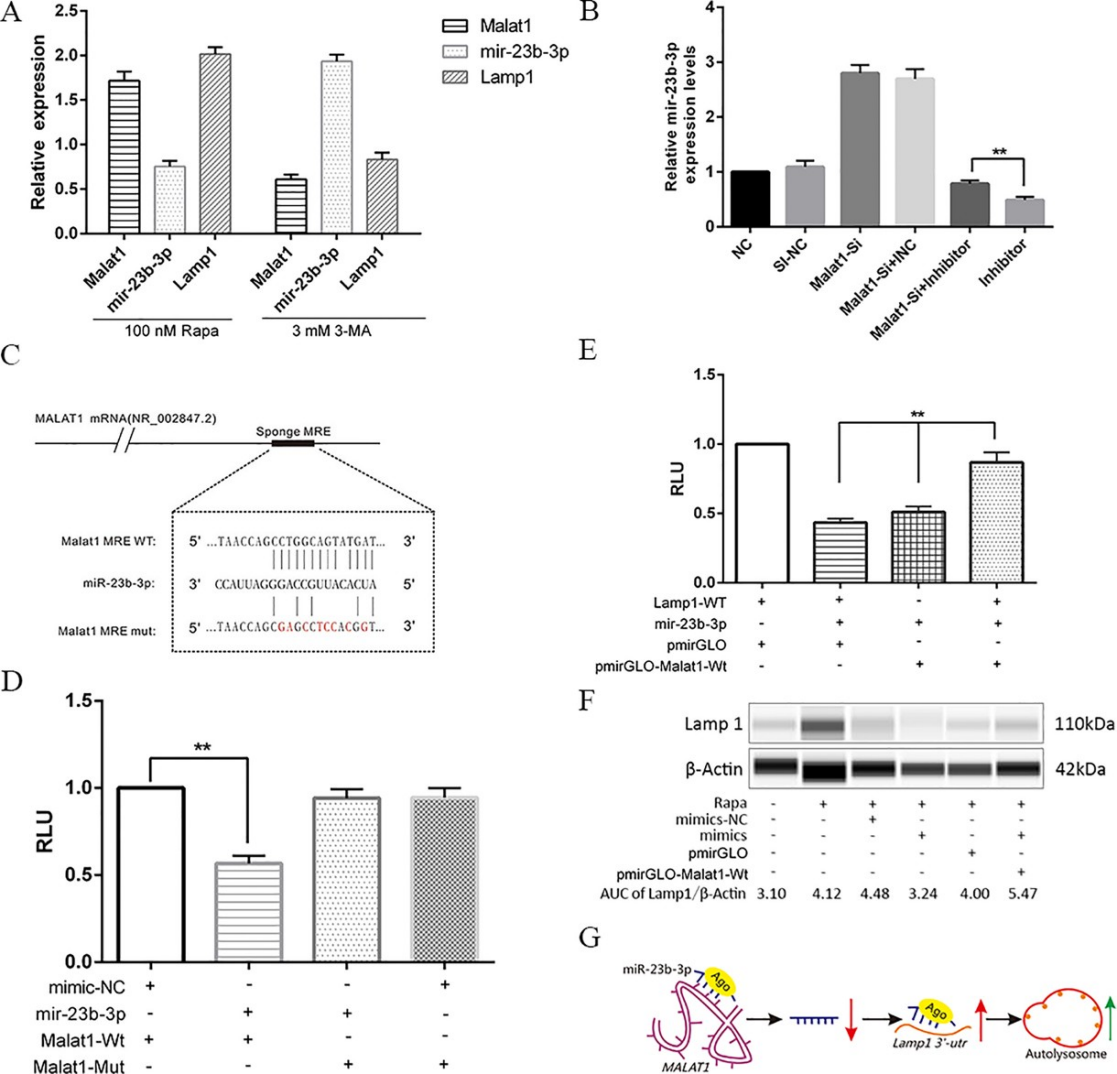
Malat1 functions as CeRNA to regulate Lamp1 expression by sponging mir-23-3p in macrophage cells. (A) RT-qPCR was performed to evaluate the expression levels of Malat1, mir-23b-3p, and Lamp1 in Raw264.7 cells respective treatment with 50 nM Rapa 2h or 3 mM 3-MA 12h (Mean±SD; n = 3). (B) RT-qPCR was used to test mir-23b-3p expression in the different groups, including NC, Si-NC, Malat1-siRNA, Malat1-siRNA+inhibitor-NC (INC), Malat1-siRNA+inhibitor, or inhibitor (Mean±SD; n = 3). (C) The bioinformatics predicted MRE of mir-23b-3p on Malat1. (D) Luciferase reporter assay was performed in 293T cells co-transfected with pmirGLO-Malat1-Wt or Mut and mimics or mimics-NC (Mean±SD; n = 3). (E) Luciferase reporter rescue experiment was conducted to confirm co-transfected pmirGLO-Malat1-Wt, pmirGLO-Lamp1-Wt and mir-23b-3p mimics effect the relative luciferase activity in 293T cells (Mean±SD; n = 3). (F) WES was used to confirm over-expression Malat1 can promote Lamp1 expression in Raw264.7 cells. (G) Graphic summary of Malat1 function in this work.

LncRNA *Malat1* has been widely reported to regulate gene expression through the sponge mechanism. *Lamp1* mediates the fusion of autophagosome with lysosomes, and is closely related to autophagy and infection immunity. *Malat1* LncRNA CeRNA network analysis and *mir-23-3p* target analysis showed that LncRNA *Malat1* may inhibit the autophagy of macrophages by release the inhibition of *mir-23-3p* on *Lamp1* through the CeRNA mechanism. Therefore, whether *malat1* promotes macrophage autophagy through CeRNA mechanism is worthy of further study. qPCR was used to analyze the differential expression of *Lamp1*, *mir-23-3p*, and *Malat1* with treatment of 100 nM RAPA or 3 mM 3-MA, and the results showed that *Lamp1* had the same expression trend as *Malat1* (**Fig 8A**). Expression of *mir-23-3p* in the control, Malat1-si, Malat1-si+Inhibitor normal control(INC), Malat1-si+inhibitor, or inhibitor groups was detected by qPCR, and the results suggested that Malat1-siRNA had promoted the upregulation of *Lamp1* (**Fig 8B**). To investigate whether the expression of mir-23-3p was regulated by lncRNA, Diana Tools (http://diana.imis.athena-innovation.gr/), RNA22, and Segal Lab (http://genie.weizmann.ac.il/) were jointly used to predict the lncRNA that might target mir-23-3p. Bioinformatics analysis revealed that *Malat1* lncRNA contained a putative 13-mer-binding motif within mir-23-3p (**Fig 8C**). *Malat1* was cloned downstream of the luciferase gene. The construct was named PmirGLO-Malat1-WT/Mut, and it was transfected with mir-23-3p mimics, inhibitor, or corresponding NC. A dual luciferase assay was conducted, and the results indicated that the luciferase activity reduced by ~40% when co-transfected with Malat1-Wt and mimics compared with the mimic-NC; however, suppression of luciferase activity was completely abolished in transfected luciferase reporter plasmid of PmirGLO-Malat1-Mut than in wild-type vector of pmirGLO (**Fig 8D**). Moreover, as expected, the rescue experiments showed that *Malat1* promoted luciferase expression in a *mir-23-3p*-dependent manner upon transient transfection with the *pmirGLO-Malat1-Wt* plasmid into Raw264.7 cells (**Fig 8E**). We further used Simple Western technology (Protein Simple, USA) to confirm that the interaction between *Malat1* and *mir-23-3p* could regulate the protein expression of LAMP1. The results showed that transient transfection with pmirGLO-Malat1-Wt plasmids significantly elevated Lamp1 expression compared with pmirGLO empty vector (**Fig 8F**). Interestingly, while knockdown of *malat1* expression (si-Malat1) compared to the si-NC group downregulated the expression of *Lamp1*, suppression of *mir-23-3p* by its inhibitor increased *lamp1* expression compared to the si-Malat1 with inhibitor NC group (**S5 Fig**). In short, autophagy stress can change the lncRNA profiles, including *Malat1*, which serves as a CeRNA to regulate *Lamp1* expression by sponging *mir-23-3p* in macrophages (**Fig 8G**).

## Discussion

It has been found that 80% of ncRNAs are lncRNAs, a class of transcripts involved in gene expression and regulatory functions [44]. Unlike miRNA, which has been broadly investigated in autophagy [45], the functions of most lncRNAs and their potential roles in autophagy are still unclear. High-throughput sequencing provides a convenient and reliable approach for researching ncRNAs at the transcription level [46,47]. With the development of sequencing technology and bioinformatics, the functions of lncRNAs in many cells and species have been described in previous studies [48–51]. However, few studies have focused on lncRNAs and their functions in the autophagy of macrophages, which plays a key role in the immune system.

Cell autophagy is precisely controlled and is highly regulated by multiple signaling pathways, orchestrated by more than 30 autophagy-related proteins organized in several functional units and many other proteins that regulate ncRNAs, including miRNAs and lncRNAs. New evidence has indicated that lncRNAs are emerging as critical regulators of autophagy and the immune process at the epigenetic and transcriptional levels in mammals in response to different stressors. Macrophages are linchpins of innate immunity, responding to invading microorganisms by initiating coordinated inflammatory and antimicrobial programs. Autophagy is a powerful weapon that macrophage cells use to defend against pathogenic microorganism infection, but its regulation in macrophages by lncRNAs is poorly defined. Here, through transcriptomic, bioinformatics, and functional studies, we focused on the specific DEL *malat1*. Systematic information about this lncRNA was acquired and the interaction between *Malat1-mir-23-3p-Lamp1* was found, suggesting that through the competitive adsorption of *mir-23- 3p*, *Malat1* releases inhibition of *Lamp1* and promotes autophagy in macrophages. These data provide a better understanding of lncRNA function in macrophages as well as a basis for further investigation into the roles and mechanisms of lncRNAs, especially *Lnc-Malat1*, in the pathogenesis of macrophages.

There have been few studies on the regulation of macrophage autophagy by lncRNAs. Mao *et al*. [52] suggested that lncRNAs may be important regulators of the lipolysaccharide-induced innate immune response in bone marrow-derived macrophages. For example, downregulation of maternally expressed 3 lncRNA eliminates mycobacteria in macrophages via autophagy [53]. Some studies have used microarrays to demonstrate that there is substantial abnormal lincRNA and mRNA expression profiles in response to pathogenic microorganisms in monocyte-derived human macrophages infected with human immunodeficiency virus or Mycobacterium tuberculosis [54]. LncRNAs are known to be involved in regulating autophagy, but sufficient data are lacking regarding their expression profile and role in autophagy in macrophages; thus, additional studies are needed to identify and clarify their roles and potential mechanisms.

In this study, RNA-Seq data were used to identify all of the potential transcripts, followed by computational analysis to determine putative lncRNAs under different autophagy stressors in Raw264.7 cells. A total of 94761 transcripts were reconstructed from the data. In comparison with 27099 transcripts (based on ESEMBL GTF file for Mouse), the number of identified transcripts, by assembled transcriptome, increased two-fold (Log_2_FC ≥ 2,94761) due to both novel isoforms of known genes as well as new genes. In total, 26282 of 94761 transcripts were predicted to be unknown transcripts and analyzed to identify putative lncRNA using credible computational methods. Finally, we obtained 1112 putative lncRNAs, including 831 large intergenic, 129 intronic, and 152 anti-sense lncRNA, in which 59 differentially expressed transcripts exhibited more than a 1.5-FC under different conditions. A total of 240 novel lncRNA sequences were found in macrophages in this work, and 8 were confirmed by qPCR in Rapa conditions.

The differential expression of lncRNA in cells can be induced by pathogens and may regulate the host response to pathogens [55]. A growing body of literature has reported the specific involvement of lncRNAs in the host cell response towards bacterial infections [56]. Viruses can hijack a host lncRNA to replicate [57]. Increasing evidence suggests that lncRNAs may regulate toll-like receptor signaling and innate immunity in APCs including macrophages [58]. However, the lncRNA profile in macrophages is still large unknown. In this study, we identified 271 novel lncRNAs in a macrophage autophagy model. Among them, there were 67 DELs in the RAPA treatment group, 37 of which were upregulated and 9 that were downregulated. Analysis of the lncRNA cis and trans target pathways showed that DELs acted as key regulators of pathways, such as the PI3K-Akt, mTOR and HIF-1α pathway. We also acquired a series of autophagy-related lncRNA-target interaction pairs for further studies.

One of the eight confirmed DELs, a newly identified functional lincRNA named *Malat1*, also known as a *Neat2*, has been recently studied in many fields. LncRNA *Malat1* is a large, infrequently spliced ncRNA that is highly conserved in mammalian evolution and widely expressed in many tissues among primates, with a homology of up to 90% between human and Mus musculus at the 3' end of the nucleotide sequence of 5 kb, suggesting that *Malat1* plays an important role in the evolution process. The full sequence of *Malat1* has a 4 highly homologous region in human and Mus musculus, suggesting that these regions are most likely functional regions of Malat1[59,60]. LncRNA *Malat1* plays a role in multiple physiological processes, such as alternative splicing, nuclear organization, and epigenetic modulation of gene expression, and it is also closely related to various pathological processes ranging from diabetes complications to cancer. It has been reported that *Malat1* can directly interact with a variety of important transcription factors to regulate gene expression, including SRSF1-3, JUN, DND1, BAF57, HuR and YAP [60]. At the same time, it was also reported that LncRNA *Malat1* expression is regulated by Ago2, but the detailed mechanism of action is unknown. Recently, LncRNA *Malat1* also positively regulates cell motility via the transcriptional and/or post-transcriptional regulation of motility-related genes [61], and modulates many genes through the sponge mechanism via different miRNAs in cancer, indicating its ability to regulate gene expression in cytoplasm [62,63]. *Malat1* has been extensively studied in pan-cancer and is reportedly an anti-oncogene, but its autophagy function in macrophage has rarely been reported.

Recently, existing research has specified that *Malat1* regulates autophagy through the sponge of mir-23-3p, which targets *HMGB2* and *ATG12* in gastric cancer [64]. In these studies, *Malat1* promoted autophagy in Raw264.7 cells. *Malat1* responded to RAPA stimulation, and was involved in the PI3K-Akt pathway in autophagy in macrophages. Interestingly, our unpublished RNA-seq data also showed that *Malat1* was significantly upregulated in macrophages infected with BacillusCalmette-Guerin (BCG) at 12 h compared to NC (data not published). To summarize, the information suggests that *Malat1* may play a crucial role in macrophages. Overexpression of *Mala1* promoted macrophage autophagy protein marker expression and autophagy flux, but blocked *Malat1* inhibition of autophagy. These results suggest that *Malat1* promotes autophagy in macrophages.

Until recently, supported lncRNAs could modulate broadly biological functions through a variety of interactions between RNA-protein, RNA-DNA, or RNA-RNA. Among them, CeRNA mechanisms were the most frequently reported. LncRNAs and miRNAs and protein complexes can associate with each other and regulate gene expression through post-transcriptional regulation. miRNAs and lncRNAs mediate dynamic regulation of gene expression, and the ternary regulatory relationship of miRNA-lncRNA-mRNA expands the central principle of genetic information and is increasingly being studied by researchers. *Malat1* functions as a sponge to regulate gene expression in many studies; however, whether *malat1* functions as a CeRNA and its new sponge miRNA and new targets are important research questions in macrophage.

In exploring the potential mechanism of *Malat1*, human ChIP-seq data from SatrBase V4 and autophagy background genes were used to construct the *Malat1* autophagy CeRNA network at first. As a result, we have obtained a better understanding of the possible functioning mode of *Malat1* in autophagy process. Malat1-miRNA was picked out from human autophagy CeRNA network to further analysis in study. Next, RNA22 software was used to explore its interaction in mouse *Malat1* sequence.

We found that *Malat1* may be as decoy of *mmu-mir-23a/b-3p*, and interaction between *malat1* and *mir-23-3p* has been confirmed in gastric cancers cells through RIP experiments [39,64]. Collectively, our work focuses on discovering new targets of *mir-23-3p* could involve in the autophagy process in macrophages. Bioinformatics analysis was performed to explore the potential targets of *mir-23-3p*. The related miRNAs of *mir-23-3p* were predicted using miRanda, TargetScan, miRDB, and DIANA databases. The minimum free energy, seed region, and miRNA recognition elements of miRNA-mRNA duplexes were calculated to choose superior candidates [40]. We found 26 mRNA may be the targets of mir-23-3p, including *Lamp1*, *ATG12*, *HMGB2*, *ORMDL1*, and *CTCF*. *ATG12* and *HMGB2* have already been reported to be involved in autophagy. We further searched for targets of mir-23-3p through document mining, and a related PPI network was constructed by STRING (https://string-db.org/cgi/input.pl). The results showed that *mir-23-3p* may be involved in the PI3K-Akt pathway. *Lamp1* was selected as a superior candidate according to gene function and human *Malat1* CeRNA network. The dual luciferase reporter assay and western blotting were used to further verify the interaction between *mir-23-3p* and *Lamp1*. The results showed that *mir-23-3p* mimics significantly reduced the luciferase activity and expression of *Lamp1* to different levels compared with the mimic-NC group. After confirming the interaction, we found that *mir-23-3p* could alternate autophagy level in macrophages. With the results of immunofluorescence, autophagy flux, and western blotting, we proved *mir-23-3p* inhibitor autophagy through targets *Lamp1* in macrophage. Ultimately, the expression of *Malat1*, *Lamp1*, and *mir-23-3p* was examined through qPCR, and we found that *Malat1* had the same expression trend as *Lamp1* but the opposite expression trend as *mir-23-3p*. Based on the dual luciferase reporter assay and rescue experiment, the interaction between *Malat1* and *mir-23-3p* was confirmed, and overexpressed *malat1* promoted *Lamp1* expression through WES. *malat1-mir-23-3p-Lamp1* regulation axis in autophagy in macrophage was confirmed. We found *malat1* responds to different autophagy stress and exhibited the capacity of sponge *mir-23-3p*, which can regulate a new crucial autophagy relative target *Lamp1*, and it took part in autophagy lysosome formation. Combined with literature analysis and pathway analysis of *mir-23-3p*, shown it may have the crucial role to regulate autophagy in macrophage through broadly affects some key genes, including *Lamp1*, *ATG12*, *HMGB2*, and *AKT1/2*. As far as we know, there is no report about the sponge regulation between *Malat1*, *mir-23-3p*, and *Lamp1* in autophagy in macrophage currently.

The phosphatidylinositol 3-kinase/AKT/mammalian target of rapamycin (PI3K/AKT/mTOR) axis is a key pathway implicated in regulating autophagy. Studies have indicated that *Malat1* can regulate autophagy through the PI3K/Akt pathway. Li X et al [65] reported that *Malat1* knockdown markedly suppressed autophagy by inhibited PI3K, Akt and p70S6K phosphorylation in HUVECs cell lines. But the mechanism of 3-MA and Malat1′s regulation of PI3K/AKT pathway on the autophagy process in Raw264.7 was uncertain.

3-Methyladenine (3-MA) blocks autophagy by inhibiting PI3K, and PI3K activity is essential for nucleation and assembly of early membrane pools in autophagosome formation. Malat1 ceRNA network analysis indicated that Malat1 can bind to mir-23-3p and participate in PI3K-AKT signaling pathway to regulate macrophage autophagy. The detection of 3-MA at mRNA level inhibited the expression of *Malat1*, which may be due to the action of 3-MA on the early stage of autophagy, and the negative feedback mechanism after the inhibition of PI3K-AKT signaling pathway reduced the expression of Malat1, increasing the expression of mir-23-3p further inhibits the PI3K-AKT signaling pathway, but this requires further experimental evidence.

In brief, we obtained a detailed lncRNA profile and autophagy-related lncRNA-mRNA pairs with different autophagy stressors in macrophages. Our results strongly suggest that *Malat1* functions as a CeRNA to regulate *Lamp1* expression by sponging *mir-23-3p* in macrophages. In this *Malat1-mir-23-3p-Lamp1* axis, *Malat1* releases inhibition of *Lamp1* and may promote autophagy in macrophages. These results provide a basis for further investigation of the roles and mechanisms of lncRNA, especially *Malat1*, in the pathogenesis of macrophages and the immune system.

## Supporting information

S1 Table RNA-seq quality control (QC) files. (DOCX)

S2 Table All LncRNA information in current work. (XLSX)

S3 Table All of the primers and Oligos in current work. (DOCX)

S1 Fig LncRNA and mRNA compare in RNA length (A), exon number (B) and ORF length (C).
(TIF)

S2 Fig Autophagy relative CeRNA network of Human Malat1, the green circle represents LncRNA malat1, the blue box represents the autophagy-related gene, and the red triangle represents the potentially bound miRNA with Malat1. (JPG)

S3 Fig PPI analysis of mir-23b-3p targets by Cytoscape through text mining from pubmed. (TIF)

S4 Fig Luciferase report unit of Lamp1 was reduced under different concentration of mir-23-3p mimics compared to PmirGLO. (TIF)

S5 Fig Lamp1 protein expression was down-regulating in condition of Malat1 was suppressed by a mix of malat1-siRNA and malat1-ASO. (JPG)

S6 Fig PCDH-Duo lentivirus vector information. (A) The Map of Double fluorescence labeling lentivirus autophagy flux detection vector and (B) Homology modeling of expression product.
(TIF)

## Abbreviations

LncRNA: long noncoding RNA
3′-UTR: 3′-untranslated region
ASO: Antisense oligonucleotide
Luc: luciferase
RLU: Relative luciferase unit
3-MA: 3-Methyladenine
RAPA: Rapamycin
STV: starvation
DEG: differentially expressed genes
DEL: differentially expressed LncRNAs
LC3: Microtubule-associated protein light chain 3
