# Supplementary material for: LncRNA expression profile during autophagy and Malat1 function in macrophages
Source: PLoS One. 2019 Aug 19;14(8):e0221104. doi: 10.1371/journal.pone.0221104 (PMC6699732; doi:10.1371/journal.pone.0221104)
Supplement: S1 Table — (DOCX) [file pone.0221104.s001.docx]

**Table 1.** QC and Mapping Information

| Sample-ID | Total Reads | Trimmed reads | Mapped Reads | Reads Sum | Base Sum | GC (%) | N (%) | Q30 (%) |
| --- | --- | --- | --- | --- | --- | --- | --- | --- |
| 3-MA | 107051086 | 91012513 | 85.02% | 53525543 | 13424104500 | 54.38 | 0.00 | 85.76 |
| RAPA | 85965992 | 73466281 | 85.46% | 42982996 | 10753640168 | 53.17 | 0.00 | 85.73 |
| NC | 87051758 | 73475863 | 84.40% | 43525879 | 10853035562 | 54.51 | 0.00 | 85.61 |
| STV | 99955706 | 78621897 | 78.66% | 49977853 | 12518161454 | 55.94 | 0.00 | 86.71 |
