# Supplementary material for: LncRNA expression profile during autophagy and Malat1 function in macrophages
Source: PLoS One. 2019 Aug 19;14(8):e0221104. doi: 10.1371/journal.pone.0221104 (PMC6699732; doi:10.1371/journal.pone.0221104)
Supplement: S3 Table — (DOCX) [file pone.0221104.s003.docx]

**I . Primers for RT-qPCR**

1. 8 DEL

| LncRNA Primer | Primer Sequence (5'-3') |
| --- | --- |
| Lnc-Malat1 | F: GTATGTAGGCCTTTGCGGGT |
|  | R: GGTTGTGCTGGCTCTACCAT |
| Lnc-Gas5 | F: CTTCTGGGCTCAAGTGATCCT |
|  | R: TTGTGCCATGAGACTCCATCAG |
| AI662270 | F: ACATCAAGAGGCAGAACCC |
|  | R: CATGCAACTCACGGACCAA |
| Lnc-IPO7 | F: GCAGAGTCAAGGAGAAGAG |
|  | R: TTCAAGCAGGCTGATCCA |
| Lnc-RRagd | F: GCTCCAGTCACACCATAG |
|  | R: TGTACCATCTGCCAGTATG |
| TCONS-00031986 | F: GTTTCCCCATAGATCTGGC |
|  | R: ATTGTGGCTGTCCCTTAGTCT |
| TCONS-00039341 | F: CTTCCAGCTCTTTACGGTGA |
|  | R: TCTGAGTCAATGAATGCCAG |
| TCONS-00060740 | F: GTAACCGAGGCATCTAGCAT |
|  | R: CACTCTTTCATCCGAATGGGTA |

1. *Mmu-Mir-23b-3p* and *Lamp1*

| Primer | Primer Sequence (5'-3') |
| --- | --- |
| *Mmu-mir-23b-3p* | RT: CTCAACTGGTGTCGTGGAGTCGGCAATTCAGTTGAGGGTAATCC |
|  | F: ACACTCCAGCTGGGATCACATTGCCAGGG |
|  | R: CTCAACTGGTGTCGTGGA |
| *U6* | F: CTCGCTTCGGCAGCACA |
|  | R: AACGCTTCACGAATTTGCGT |
| *Lamp1* | F: GCCCTGGAATTGCAGTTTGG |
|  | R: TGCTGAATGTGGGCACTAGG |

**II. Dual Luciferase Report Plasmid Primer**

| Primer Name | Primer Sequence (5'-3') |
| --- | --- |
| pmirGLO-Lamp1-Wt | F: G*GCTAGC*CACCAGAGATGCACAGGGGCC |
|  | *R: GCGTCGAC*CTTAAGTGTGCAGCCTAACC |
| pmirGLO-Lamp1-Mut | F: G*GCTAGC*CACCAGAGATGCACAGGGGCC |
|  | *R: GCGTCGAC*GTGAGCTGTGATAAAGCAGATTTTAA |
| pmirGLO-Malat1-Wt | F: G*GCTAGC*CCTGAATGTCTTTTAGAG |
|  | R: GC*GTCGAC*CTTATCTGTCAACAGCAGTC |
| pmirGLO-Malat1-Mut | F: G*GCTAGC*CCTGAATGTCTTTTAGAG |
|  | R: GC*GTCGAC*TTCCCTTAGACTGGCTGGTTATGACTC |

**III. PCDH-Duo-Malat1**

| Primer.Name | Primer Sequence (5'-3') |
| --- | --- |
| PCDH-Duo-Malat1 | F: CGGAATTCCCTGAATGTCTTTTAGAGGG |
|  | R: CGGGATCCCTTATCTGTCAACAGCAGTC |

**IV. Malat1 LncRNA siRNA and ASO oligo**

| Name | Sequence (5'-3') |
| --- | --- |
| siRNA-NONMMUT021455_001 | GGAAAGTTAGACAAGAAGA |
| siRNA-NONMMUT021455_002 | GGCTCATCTTATTATAGTA |
| siRNA-NONMMUT021455_003 | CAGACTTCACCTACAGTAA |
| ASO-NONMMUT021455_001 | GGAGAAAGAGCGAAGTGAAG |
| ASO-NONMMUT021455_002 | GCAGCAGAATGCAGAGGAAG |
| ASO-NONMMUT021455_003 | CCTTGCAAGTCATGACAAAC |

NONMMUT021455 is NONCODE database ID of *Malat1* LncRNA
