# Supplementary figures and images for: LncRNA expression profile during autophagy and Malat1 function in macrophages
Source: PLoS One. 2019 Aug 19;14(8):e0221104. doi: 10.1371/journal.pone.0221104 (PMC6699732; doi:10.1371/journal.pone.0221104)

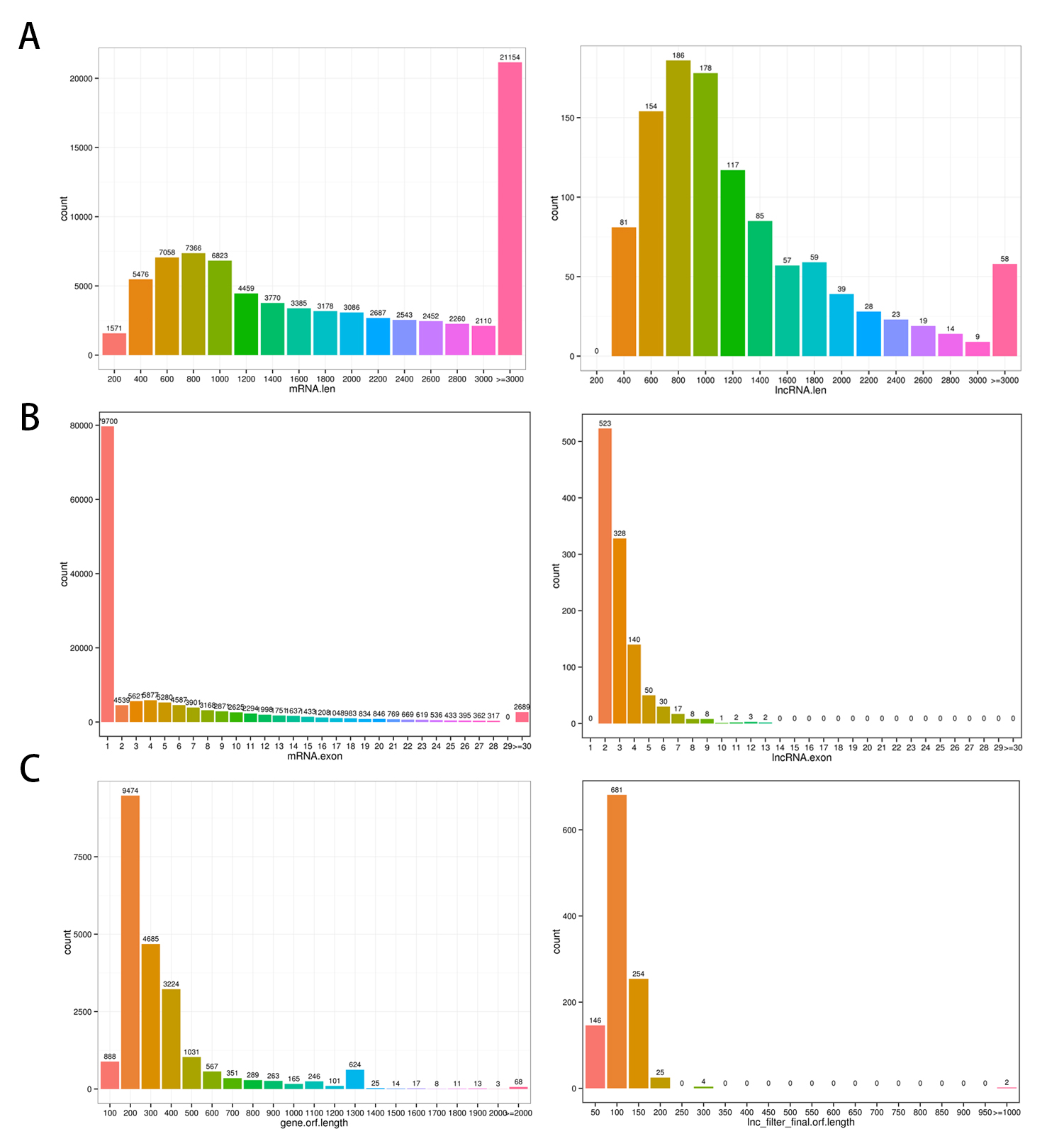

Supplement: S1 Fig — LncRNA and mRNA compare in RNA length (A), exon number (B) and ORF length (C). (TIF) [file pone.0221104.s004.tif]

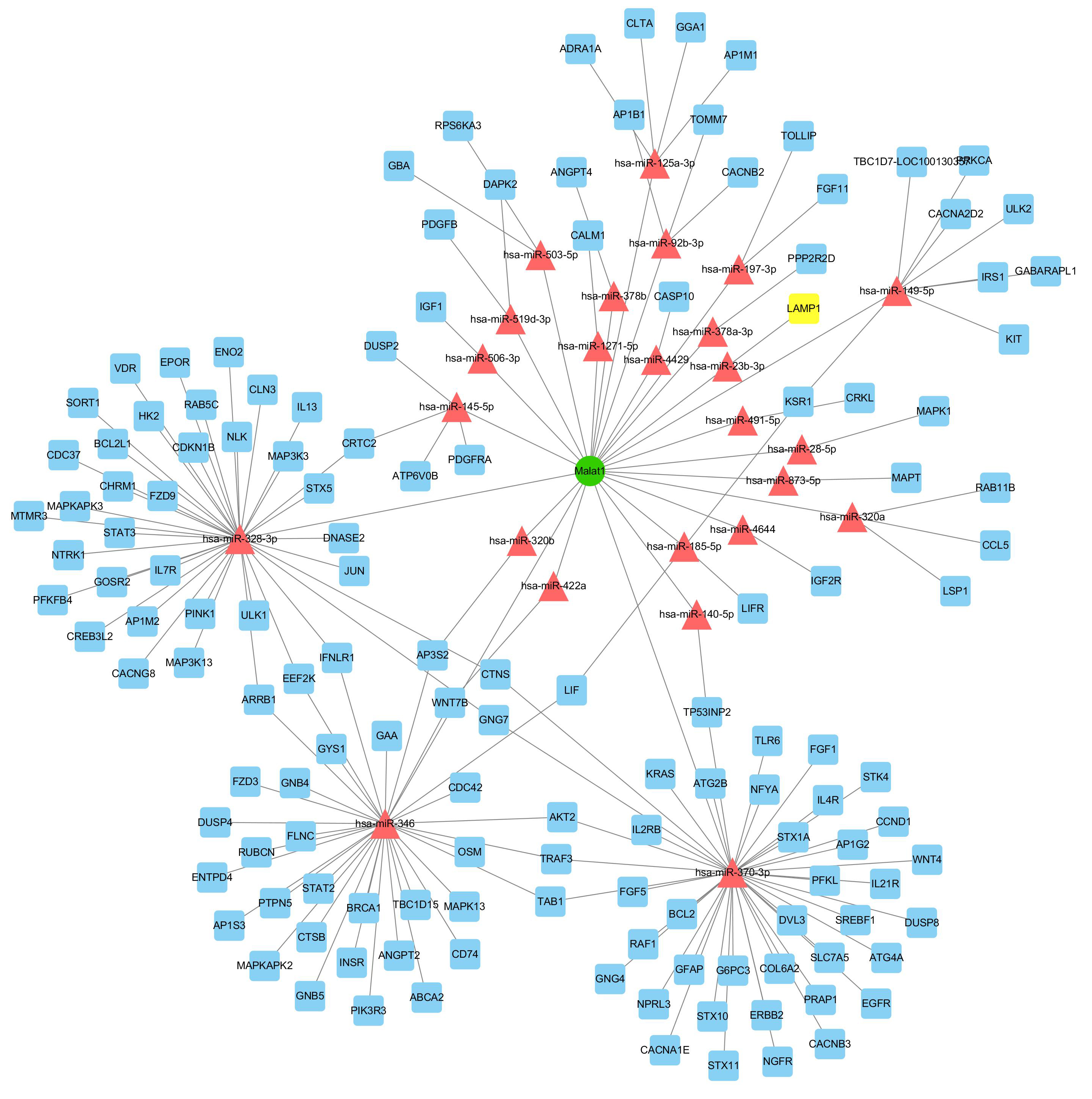

Supplement: S2 Fig — (JPG) [file pone.0221104.s005.jpg]

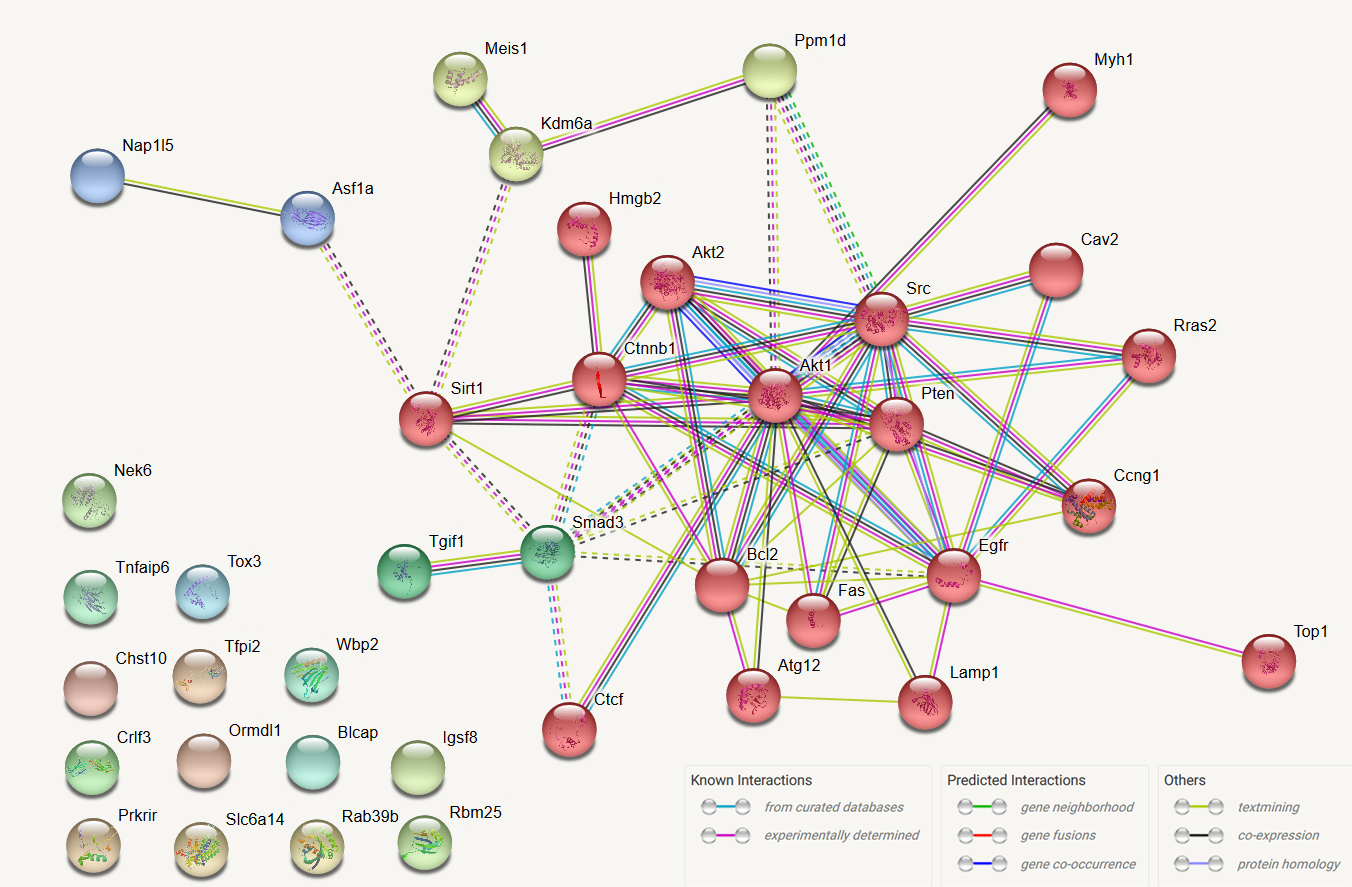

Supplement: S3 Fig — (TIF) [file pone.0221104.s006.tif]

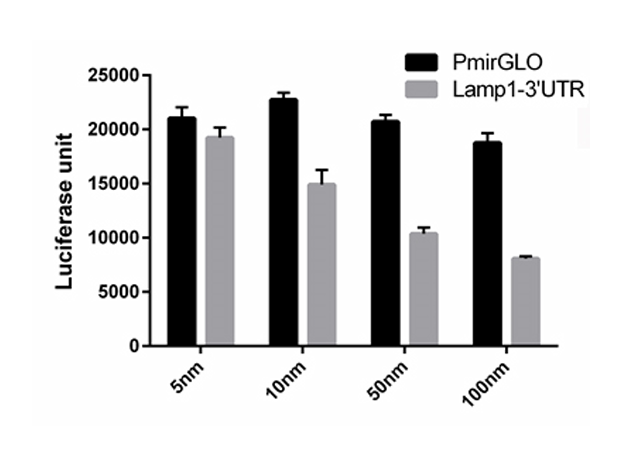

Supplement: S4 Fig — (TIF) [file pone.0221104.s007.tif]

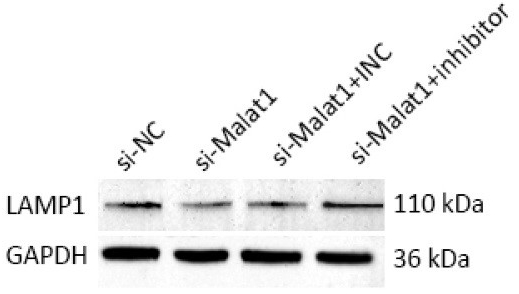

Supplement: S5 Fig — (JPG) [file pone.0221104.s008.jpg]

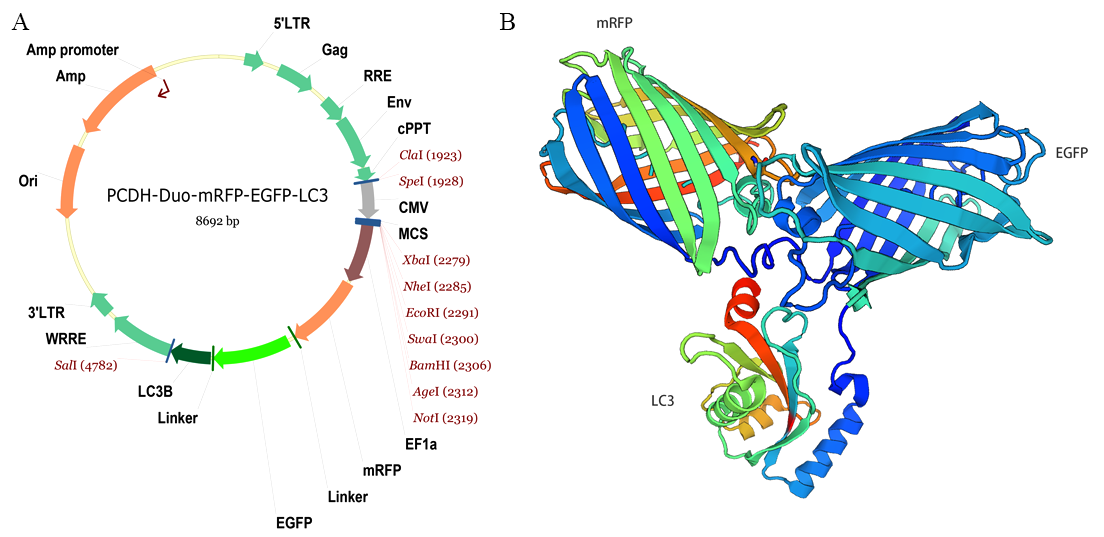

Supplement: S6 Fig — (A) The Map of Double fluorescence labeling lentivirus autophagy flux detection vector and (B) Homology modeling of expression product. (TIF) [file pone.0221104.s009.tif]
